# Genetic evidence for PARP1 trapping as a driver of PARP inhibitor efficacy in *BRCA* mutant cancer cells

**DOI:** 10.1093/nar/gkaf1398

**Published:** 2025-12-29

**Authors:** Jonathan Ribeiro, Kjetil Hansen, Lotte van Beek, Christopher Stubbs, Joan Frigola, Sara Talbot, Sabrina Bentouati, James Hall, Paul W G Wijnhoven, Josh Armenia, Marianne Schimpl, Josep V Forment, Mark R Albertella, Mark J O’Connor, Giuditta Illuzzi

**Affiliations:** OTD Bioscience, Early Oncology, AstraZeneca, CambridgeCB20AA, United Kingdom; Discovery Sciences, R&D, AstraZeneca, CambridgeCB20AA,United Kingdom; Discovery Sciences, R&D, AstraZeneca, CambridgeCB20AA,United Kingdom; Discovery Sciences, R&D, AstraZeneca, CambridgeCB20AA,United Kingdom; Oncology Data Science, Oncology R&D, AstraZeneca, CambridgeCB20AA,United Kingdom; OTD Bioscience, Early Oncology, AstraZeneca, CambridgeCB20AA, United Kingdom; OTD Bioscience, Early Oncology, AstraZeneca, CambridgeCB20AA, United Kingdom; OTD Bioscience, Early Oncology, AstraZeneca, CambridgeCB20AA, United Kingdom; OTD Bioscience, Early Oncology, AstraZeneca, CambridgeCB20AA, United Kingdom; Oncology Data Science, Oncology R&D, AstraZeneca, CambridgeCB20AA,United Kingdom; Discovery Sciences, R&D, AstraZeneca, CambridgeCB20AA,United Kingdom; OTD Bioscience, Early Oncology, AstraZeneca, CambridgeCB20AA, United Kingdom; OTD Bioscience, Early Oncology, AstraZeneca, CambridgeCB20AA, United Kingdom; OTD Bioscience, Early Oncology, AstraZeneca, CambridgeCB20AA, United Kingdom; OTD Bioscience, Early Oncology, AstraZeneca, CambridgeCB20AA, United Kingdom

## Abstract

Clinically approved PARP inhibitors (PARPi) have shown significant efficacy as monotherapy in homologous recombination repair (HRR)-deficient cancers. PARPi suppress PARP enzymatic activity but can also induce PARP trapping onto DNA lesions, and there is an ongoing debate on which of these properties is key in determining their clinical efficacy as single agents. In this study, we found that the enzymatic activity of PARP1 is dispensable for the survival of a *BRCA1* mutant (*BRCA1*m) breast cancer model. However, PARP1 expression is necessary for the efficacy of PARPi in this model, supporting the importance of PARP1 trapping. We also identified and characterised a PARP1 mutation resulting in loss of the enzymatic inhibition and trapping activity of the PARP1-selective inhibitor, saruparib. However, the same mutation increased the trapping ability of other PARPi, namely veliparib and olaparib, without enhancing their enzymatic inhibition activity, a change that led to an increase in efficacy in this *BRCA1*m model. Together, these data suggest that PARP1 trapping, and not only its enzymatic inhibition, is a key driver for PARPi effectiveness in *BRCA1*m cancer cells.

## Graphical Abstract

**Graphical Abstract:**
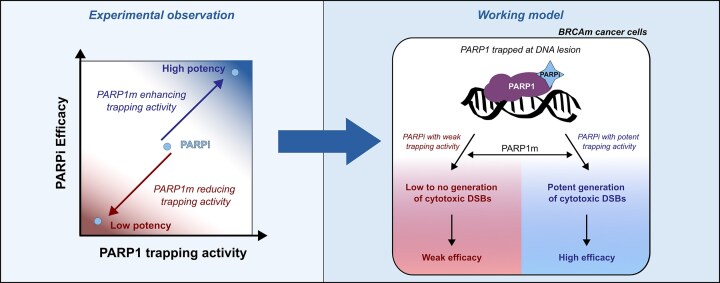

## Introduction

PARP1 is an evolutionary conserved protein playing a central role as a sensor of DNA damage [1], rapidly binding to a large spectrum of DNA lesions, including DNA single- and double-strand breaks and DNA structures such as R-loops [1, 2]. PARP1 orchestrates the repair of these lesions through its enzymatic activity by synthesising poly-ADP-ribose (PAR) chains on target proteins, a process known as PARylation [3–5]. This post-translational modification requires nicotinamide adenine dinucleotide (NAD^+^) as a substrate that can be recycled by glycohydrolysis of PAR chains, a process mainly catalysed by the PARG glycohydrolase [6].

PARP1 PARylation activity can be inhibited using small-molecule inhibitors. The development of these inhibitors (PARPi) began over forty years ago [7] and the profiling of subsequent generations of PARPi led to the identification of two distinct mechanisms of action potentially explaining their cytotoxicity. First, inhibition of the enzymatic activity of PARP1 leads to the disruption of the PARylation-dependent signalling involved in the repair of DNA lesions [5]. Second, in addition to PARP1 enzymatic inhibition, PARPi can cause PARP1 trapping on certain DNA lesions, impeding their repair and ultimately leading to the generation of highly cytotoxic DNA double-strand breaks in replicating cells [8–10].

Structural studies of the physiological catalytic cycle of PARP1 have shown that binding to a DNA lesion propagates a conformational change involving the DNA binding domains and catalytic domain, thereby regulating PARP1 catalytic activity [11, 12]. This conformational change alters the dynamics of the autoinhibitory (HD) domain of PARP1, allowing the catalytic domain to bind to NAD^+^, thus initiating PARylation activity. PARPi compete with NAD^+^ for the binding to the catalytic domain, explaining how they prevent the enzymatic activity of PARP1 [13]. Moreover, in a mechanism termed reverse allostery, binding of a PARPi to the catalytic domain of PARP1 transfers another conformational change from the catalytic domain to the DNA binding domains of PARP1, leading to increased affinity for DNA and the associated PARP1 trapping onto the DNA lesion [8, 14, 15].

A subset of ovarian, breast, pancreas and prostate cancers exhibit biallelic mutations in genes involved in the homologous recombination repair (HRR) pathway, most notably, the *BRCA1* or *BRCA2* genes [16, 17]. These mutations can result in a complete loss of function of the HRR genes or expression of hypomorphic HRR alleles [18–22]. The seminal discovery of the ability of early PARP small molecule inhibitors, or *PARP1* silencing, to selectively kill BRCA1- or BRCA2-deficient cells [23, 24], kickstarted the development of clinically approved PARPi, which have shown significant efficacy as monotherapy in HRR-deficient cancers [25]. Subsequent profiling of these PARPi has demonstrated that their PARP1 trapping abilities strongly correlate with their cytotoxic potencies in BRCA-deficient cells, independent of their ability to inhibit PARP catalytic activity [8, 9]. Collectively, these works have introduced the notion that in addition to potent enzyme inhibition, the therapeutic efficacy of PARPi may be driven by their trapping ability [26]. This concept was further reinforced by reports showing that the efficacy of PARPi requires PARP1 DNA binding and a functional reverse allostery [14, 27, 28]. However, a recent report has posited that PARP trapping may not be necessary for the therapeutic efficacy of PARPi [29], indicating that the key properties of PARPi driving their efficacy remain a subject of ongoing debate.

Since there are no PARPi that exclusively display potent PARP1 trapping activity without PARylation inhibition, demonstrating the properties of PARPi that drive therapeutic efficacy is challenging. In the absence of such compounds, we sought to use a genetic approach to delineate the properties of PARPi underlying efficacy in *BRCAm* cancer cells. By modulating the trapping capabilities of saruparib and veliparib, respectively potent and weak PARP1 trappers [8, 30], our results indicate that PARP1 trapping is a key component of PARPi effectiveness in *BRCA1*m cancer cells.

## Materials and methods

### Small molecule inhibitors

All PARP inhibitors, saruparib, olaparib, veliparib, and talazoparib, ceralasertib and carboplatin were synthesised by AstraZeneca [31, 32]. PARG (PDD 00017273, Tocris Bioscience, #5952) and NAMPT (CHS-828, Tocris Bioscience, #6753) inhibitors were purchased. All compounds were dissolved in DMSO at 10 mM stock concentration, and stored protected from light.

### Cell lines and cell culture

All cell lines were grown under standard conditions (37°C and 5% CO2). SUM149PT cells were maintained in Ham’s F-12 medium (Sigma-Aldrich, # N3520) supplemented with 5% FBS, 1X GlutaMAX (Gibco, #35 050 038), 10 µg/ml insulin (Sigma-Aldrich, #I9278) and 0.5 µg/ml hydrocortisone (Sigma-Aldrich, # H0396). HEK293T cells were cultured in DMEM medium (ThermoFisher Scientific, # 11 965 092) supplemented with 10% FBS. DLD-1 cells were cultured in RPMI 1640 medium (ThermoFisher Scientific, #11 835 030) supplemented with 10% FBS and 1X GlutaMAX (Gibco, #35 050 038). A549 *PARP1/PARP2* KO cells were cultured in Ham's F-12K medium (ThermoFisher Scientific, # 21 127 022) supplemented with 10% FBS and 1X GlutaMAX (Gibco, #35 050 038). Cell lines were obtained from ATCC, Horizon Discovery, or BioIVT; authenticated by the AstraZeneca cell bank using short tandem repeat analysis using CellCheck (IDEXX Bioanalytics); and validated free of Mycoplasma contamination using the STAT-Myco assay (IDEXX Bioanalytics).

LSE SUM149PT cells were generated by first cultivating parental SUM149PT cells in presence of 3 µM saruparib for two weeks. Then, cells were cultivated in presence of 9 µM saruparib for two weeks, and 20 µM saruparib for two other weeks. Individual saruparib-adapted clones were subsequently isolated, amplified and maintained under standard culture conditions and with 1 µM saruparib.

### Cell line engineering

To target the *PARP1* locus in SUM149PT and HEK293T cells, sgRNAs (PARP1_A, 5′-CACGCTCTATCTTAAAGATC-3′, PARP1_B, 5′-AGTGACAGGTGTGTACATAC-3′, PARP1_C, 5′-GGGACTTTTCCATCAAACAT-3′) and Cas9 (Integrated DNA Technologies, #1 081 058) were diluted at 60 and 300 nM respectively in 150 µL of Opti-MEM (Gibco, #31 985 062) containing 3.6 µL of Cas9^+^ Reagent (Invitrogen, #CMAX00001). The mixture was let to incubate at room temperature for 5 min. Then, 7.5 µL of CrisprMAX Transfection reagent (Invitrogen, #CMAX00001) and 143 µL of Opti-MEM were added to the mixture and the reaction mix was incubated at room temperature for 20 min. Next, 600 µL of a cell suspension containing 240 000 cells in their culture medium was mixed with the sgRNA/Cas9 transfection mixture. Cells were transferred in a well of a 24-well plate and incubated at 37°C, 5% CO_2_. Media was changed 24-h post-transfection and cells were allowed to recover. Cells were seeded in 96-well plates as single cells, and individual clones were subsequently analysed by western blot to assess PARP1 loss of expression.

A549 PARP1/PARP2 KO cells were generated as described in a prior study [30].

*BRCA1* reversion SUM149PT cells were generated by infecting Cas9-expressing SUM149PT cells with *BRCA1* gRNA (5′-CCAAAGATCTCATGTTAAG-3′, [33]) followed by PARPi selection and clonal expansion. In short, two oligonucleotides, 5′-CACCGCCAAAGATCTCATGTTAAG-3′ and 5′-AAACCTTAACATGAGATCTTTGGC-3′ representing the abovementioned gRNA, were annealed and then inserted into the pKLV2-U6gRNA(Bbsl)-PCKpuro2AZsG-W plasmid digested with the Bbs1 (#R0539S, New England Biolabs) restriction enzyme. Lentiviruses were generated by transfecting (Lipofectamine LTX, # A12621, Invitrogen) HEK293 cells with the abovementioned gRNA-contained plasmid, the psPAX2 packaging plasmid and the mMD2.G VSV-G envelope plasmid. Three days following transfection, supernatants containing virus were collected and 500 µL/well supernatant plus 8 µg/mL polybrene were used to infect 200 000 SUM149PT-Cas9 cells (6 well plates). Three days following transduction, cells were put under selection pressure through continuous exposure to 3 µmol/L olaparib for several weeks. Individual clones were subsequently isolated, expanded and validated by western-blot indicating the expression of a BRCA1 protein with a molecular weight consistent with reversion event (approximatively 200 kDa). TIDE (Tracking of Indels by Decomposition) analysis confirmed the absence of the parental *BRCA1* 2288delT allele and presence of a prominent -13 deletion from cut site.

### TIDE (Tracking of Indels by Decomposition) analysis of BRCA1 reversion

Genomic DNA was extracted from successfully isolated clones by lysis with DirectPCR (#302-C, Viagen) and Proteinase K (#19 131, Qiagen) lysis mix. Cells were lysed for 10 min in 40 µL of lysis solution per well of a 96-well plate whilst agitated on a plate shaker. Complete cell lysis was confirmed via microscope then lysates were transferred to a 96-well PCR plate (#3753, Corning), sealed, and incubated on a thermal cycler to complete extraction of genomic DNA.

Region of interest on the *BRCA1* gene was amplified by PCR using Q5 Master Mix (#M0492S, NEBiolabs) and the following forward and reverse primers (5′-CAAATGCCAGTCAGGCACAG-3′ and 5′-CCCAGAGTGGGCAGAGAATG-3′). Resulting product was then purified using QIA Quick PCR Purification kit (#28 106, Qiagen) and sent for Sanger sequencing via Eurofins. Sequencing files were analysed using the TIDE web tool (http://shinyapps.datacurators.nl/tide/) [34] to compare sequences to wild-type SUM149PT cells, identifying a prominent −13 deletion from cut site.

### Plasmids

Before use, all plasmids were validated by Sanger sequencing. First, pIRES-H2B-GFP was generated by cloning H2B-GFP cDNAs into pIRES (Takara Bio, # 631 605), between XbaI and NotI sites. Then, pPARP1 (WT or L777P)-IRES-H2B-GFP plasmids were generated by cloning PARP1 (WT or L777P) cDNAs into the pIRES-H2B-GFP plasmid, between XhoI and MluI sites. Expression plasmids for Halo-tagged PARP1 variants were generated by cloning PARP1 (WT or L777P) cDNAs into the pHTN CMV-neo vector (Promega, # G7721), between XbaI and ApaI sites.

Expression vectors for recombinant CAT PARP1 purification were generated as follow: pFastBac1 plasmids for baculovirus generation and subsequent protein expression were assembled by automated Golden Gate cloning of mammalian codon-optimised gene blocks (GeneArt, ThermoFisher) of PARP1(662–1011) and PARP1(662–1011)[L777P] as published [35].

### Western blot

Cell were lysed in SDS buffer (1% SDS, 10 mM Tris–HCl pH 7.5, 100 mM NaCl, 2mM MgCl_2_) complemented with protease inhibitors cocktail (Roche, # 11 873 580 001), 1 µM olaparib, 1 µM PARGi (PDD 00017273, Tocris Bioscience, #5952), 3 U/mL DNAse I-XT (New England Biolabs, # M0570), and 25 U/mL Benzonase (Merk, #70746–3) and lysates were incubated at room temperature for 10 min. Extracts were spun at 16,200 g for 10 min, supernatants were collected and analysed by western blot.

Protein samples were supplemented with LDS buffer (Themo Fisher Scientific, #NP0007) and Sample Reducing Agent (Themo Fisher Scientific, # NP0004), boiled for 5 min at 95°C and resolved by polyacrylamide gel electrophoresis on NuPAGE 4–12%, Bis–Tris, Mini Protein gels (ThermoFisher Scientific, # NP0322BOX) in NuPAGE MOPS SDS Running Buffer (Themo Fisher Scientific, #NP0001). Separated proteins were transferred onto 0.2 μm nitrocellulose membranes (ThermoFisher Scientific, #IB301001) using the iBlot 2 transfer system (ThermoFisher Scientific, #IB21001). Transfer was set at 20V for 1 min, then 23V for 4 mins and 25V for 5 min. After transfer, membranes were incubated for 1 h in blocking buffer (Tris-buffered saline, 0.05% Tween 20, 5% non-fat dry milk). Then, membranes were incubated ON at 4°C in blocking buffer with the following antibodies: anti-PARP1 (Cell Signaling Technology, #46D11, 1:1,000 dilution), anti-BRCA1 (Merk, clone MS110, #OP92, 1:500 dilution), anti-MAR/PAR (Cell Signaling Technology, #, 1:1,000 dilution), anti-HaloTag (Promega, # G9281, 1:1,000 dilution), anti-GAPDH (Cell Signaling Technology, #14C10, 1:5,000 dilution), anti-VINCULIN (Sigma-Aldrich, # V9131, 1:4,000 dilution), anti-PARP2 (Active Motif, #39 743, 1:1,000 dilution). Membranes were washed with TBS + 0.05% Tween 20 and then incubated for 1 h 30 min at RT with the following secondary antibodies diluted in blocking buffer: anti-rabbit IgG, HRP-linked (Cell Signaling Technology, #7074, 1:5,000 dilution) and anti-mouse IgG, HRP-linked (Cell Signaling Technology, #7076, 1:5,000 dilution). Membranes were then washed with TBS + 0.05% Tween 20. Then, membranes were incubated with SuperSignal West Dura Extended Duration Substrate (ThermoFisher Scientific, #34 075). Acquisition of the chemiluminescent signal was done using a ChemiDoc Imaging system (Bio-Rad).

### Colony formation assay

Cells were seeded in 24-well plates, 1 000 SUM149PT cells/well, 900 DLD1 wild-type cells/well or 1 500 DLD1 *BRCA2*^-/-^ cells/well. PARPi were dispensed by an automated digital D300 HP dispenser (Tecan) in titration dilutions, each concentration was tested in triplicate and DMSO was used as untreated control. Plates were incubated at 37°C, 5% CO_2_ for 9 days (SUM149PT and DLD1 wild-type cells), or 14 days (DLD1 *BRCA2*^-/-^ cells), to allow colony formation. Cells were then fixed and stained with Blue-G-250 brilliant blue (Sigma, #B8522–1EA, reconstituted in 25% (v/v) methanol and 5% (v/v) acetic acid) for 20 min then thoroughly washed with dH2O. Plates were scanned with GelCount (Oxford OPTRONIX). Colonies were analysed by total optical density measured with ImageJ software, using a 24-well plate ROI mask. Data analysis was performed by normalisation on the vehicle treated of the respective plate set as 1. Data were plotted and IC50s were calculated with GraphPad Prism software.

To target the *PARP2* locus in SUM149PT cells, *PARP2*-targeting sgRNAs (PARP2_A, 5′- AGAUCGAGAAAAGUUUGAGA-3′, PARP2_B, 5′- AAAAUAUGAUAUGCUACAGA-3′, PARP2_C, 5′- GAGAGUUACCUGAGUAUUGG-3′) or non-targeting sgRNA (Invitrogen, #A35526) and Cas9 (Integrated DNA Technologies, #1 081 058) were diluted at 60 nM and 300 nM respectively in 150 µL of Opti-MEM (Gibco, #31 985 062) containing 3.6 µL of Cas9^+^ Reagent (Invitrogen, #CMAX00001). The mixture was let to incubate at room temperature for 5 min. Then, 7.5 µL of CrisprMAX Transfection reagent (Invitrogen, #CMAX00001) and 143 µL of Opti-MEM were added to the mixture and the reaction mix was incubated at room temperature for 20 min. Next, 600 µL of a cell suspension containing 240 000 cells in their culture medium was mixed with the sgRNA/Cas9 transfection mixture. Cells were transferred in a well of a 24-well plate and incubated at 37°C, 5% CO_2_. Media was changed 24 h post-transfection and cells were allowed to recover. Eight days after transfection, cells were harvested and seeded in 24-well plates, 1000 SUM149PT cells/well. Then, cells were processed for the colony formation assay as described earlier.

### PARP trapping assay

SUM149PT cells were plated into 96-well plates (Revvity, #6 055 302). PARPi were dispensed by an automated digital D300 HP dispenser (Tecan) in titration dilutions, each concentration was tested in duplicate. Then, Methyl methanesulfonate (MMS; ThermoFisher Scientific, #H55120.06) was added at final 0.005% and cells were incubated for 4 h at 37°C, 5% CO_2_. Cell media was removed and a pre-extraction step was performed for 6 min at 4°C with cold cytoskeleton (CSK) buffer (10 mM PIPES pH 6.8, 300 mM sucrose, 200 mM NaCl, 3 mM MgCl2) supplemented with 0.3% Triton X-100. Cells were then fixed with 4% PFA (ThermoFisher Scientific, # J19943.K2) for 15 min at RT; washed with PBS then fixed a second time with ice-cold methanol for 30 min at −20°C. Cell were washed with PBS then incubated in blocking solution (PBS, 0.5% Bovine serum albumin, 0.2% gelatin from cold water fish skin (Sigma-Aldrich, #G7765)) for 30 min at 4°C. Immunostaining of PARP1 or PARP2 was performed by adding the primary antibody (PARP1 antibody, Sigma, clone 3G4, WH0000142M1; PARP2 antibody, Active Motif, #39 743) diluted 1:1 000 in blocking solution and incubated overnight at 4°C, followed by incubation with secondaries goat anti-rabbit AlexaFluor 488 and goat anti-mouse AlexaFluor 594 antibodies (1:2000 each) in blocking solution for 1 h and 30 min at room temperature. DNA was counterstained with DAPI (Thermo Fisher Scientific, #D1306, diluted 1 μg/mL in PBS). The nuclear fluorescence intensities of PARP1 and PARP2 were acquired and analysed using CellInsight CX5 HCS Platform (Thermo Fisher); dose–response curves were generated with GraphPad Prism Software.

PARP trapping on DLD-1 cells was performed as described previously but without using MMS, and by incubating DLD-1 cells with PARPi for 72h. Image acquisition and analysis was done with the scanR high content screening platform (Olympus, Evident). 20x objectives were used for image acquisition; 25–36 fields were acquired per well.

PARP1 trapping with transfected HEK293T *PARP1* KO was performed as follow. Cells were first transfected with pHTN-PARP1 WT or pHTN-PARP1 L777P vectors using the PolyFect Transfection Reagent (Qiagen, # 301 105) and following the manufacturer’s instructions. Twenty-four hours post-transfection, media was changed. Then, cells were exposed to PARPi and to the HaloTag R110Direct ligand (Promega, #G3221, 1:1000 dilution). Cells were then incubated overnight at 37°C, 5% CO_2_. The next day, cells were exposed to MMS (ThermoFisher Scientific, #H55120.06) and incubated for 3 h. Then, the pre-extraction step was performed as above except with a CSK buffer supplemented with 0.5% Triton X-100. From that step, cells detach from the culture dishes and are treated as a cell suspension. Cells were then washed with PBS-B (2% BSA in PBS) and fixed with 4% PFA (ThermoFisher Scientific, # J19943.K2) for 10 min at RT. Cell were washed with PBS-B and DNA was counterstained with DAPI (Thermo Fisher Scientific, #D1306, diluted 1 μg/mL in PBS-B). Cells were dispatched in imaging-quality 96-well plates (CellVis, #P96-1.5P) and plates were spun at 300 g for 1 min. Imaging and image analysis were performed using the scanR high-content screening platform (Olympus). Dose–response curves were generated with GraphPad Prism Software.

### DNA damage and cell-cycle analysis by immunofluorescence

SUM149PT cells were plated at a density of 10,000 cells per well into 96-well (Revvity, #6 055 302). PARPi were dispensed by an automated digital D300 HP dispenser (Tecan) in titration dilutions, each concentration was tested in duplicate. Plates were incubated for 72 h at 37°C, 5% CO_2_. Cell media was removed and a pre-extraction step was performed for 6 min at 4°C with cold cytoskeleton (CSK) buffer (10 mM PIPES pH 6.8, 300 mM sucrose, 200 mM NaCl, 3 mM MgCl2) supplemented with 0.3% Triton X-100. Cells were then fixed with 4% PFA (ThermoFisher Scientific, # J19943.K2) for 15 min at RT; washed with PBS then fixed a second time with ice-cold methanol for 30 min at −20°C. Cell were washed with PBS then incubated in blocking solution (PBS, 0.5% Bovine serum albumin, 0.2% gelatin from cold water fish skin (Sigma-Aldrich, #G7765)) for 30 min at 4°C. Cells were then incubated with the primary antibody, overnight at 4°C (γH2AX, Cell Signaling Technology, #9718, diluted 1:1 000), followed by secondary antibody goat anti-rabbit AlexaFluor 488 (Thermo, #A21099, 1:2,000) and DAPI (Thermo Fisher Scientific, #D1306, diluted 1 μg/mL in PBS). The nuclear fluorescence intensities of γH2AX and DAPI were acquired and analysed using CellInsight CX5 HCS Platform (Thermo Fisher); γH2AX dose–response curves were generated with GraphPad Prism Software. Cell cycle distributions were generated with GraphPad Prism Software using the fluorescence intensity of DAPI.

Micronuclei and gH2AX analysis on DLD-1 cells was performed as follow. Cells were seeded in imaging-quality 96-well plates (CellVis, #P96-1.5P). Compounds were added using a D300 HP dispenser (Tecan) from compound stocks dissolved in DMSO, in duplicate for each condition. Plates were incubated for 72 h. Cells were washed and fixed in 4% paraformaldehyde (PFA) for 15 min at RT and then permeabilised in PBS + 0.2% Triton X-100 for 10 min at RT. Blocking was performed using PBG: 0.5% BSA + 0.2% gelatin from cold water fish skin (Sigma, cat no. G7765) in PBS for 1 h at RT. Cells were then incubated with primary antibody overnight at 4°C (gH2AX, Merck Millipore, #05–636, diluted 1:5,000), followed by secondary antibody (anti-mouse, Alexa Fluor 594, Thermo, #A21099, 1:2 000) and DAPI (ThermoFisher, #D1306, diluted 1 µg/ml) for 30 min at RT. Image acquisition and analysis was done with the scanR high content screening platform (Olympus, Evident). 40x objectives were used for image acquisition; 25–36 fields were acquired per well. For micronuclei analysis an image analysis protocol generated with cellSens (Evident) was used.

### Immunofluorescence analysis of PARylation

PARylation inhibition assay was performed as follow. SUM149PT cells were plated at a density of 20 000 cells per well into 96-well plates (Revvity, #6 055 302). Forty-eight hours later, PARPi were dispensed by an automated digital D300 HP dispenser (Tecan) in titration dilutions, each concentration was tested in duplicate. After 3 h incubation with PARPi, H_2_O_2_ (Camlab Chemicals, #1 110 471) was added at final concentration of 10 mM and incubated for 5 min in culture conditions. Cells were then fixed in ice-cold methanol for 30 min at −20°C and processed for immunostaining with primary antibody Poly/Mono-ADP Ribose (Cell Signaling Technology, #83 732, 1:1 000) followed by secondary goat anti-rabbit AlexaFluor-488 antibody (Invitrogen, #A-11008, 1:2,000); nuclei were counterstained with DAPI (Thermo Fisher Scientific, #D1306, diluted 1 μg/mL in PBS). The nuclear PAR fluorescence intensity (F.I.) was acquired and analysed using CellInsight CX5 HCS Platform (Thermo Fisher Scientific); dose–response curves were generated with GraphPad Prism software.

PARylation activity of wild-type and mutant PARP1 was assessed as follow. A549 *PARP1/PARP2* KO cells were first transfected with pPARP1 (WT or L777P) -IRES-H2B-GFP vectors using the polyethylenimine (PEI, Polysciences, #23966–1) transfection method. Forty-eight hours post-transfection, cells were exposed to 10 mM H_2_O_2_ (Camlab Chemicals, #1 110 471) and incubated for 5 min or exposed to 0.01% MMS (ThermoFisher Scientific, #H55120.06) and incubated for 4 h at 37°C, 5% CO_2_. Cells were then fixed in ice-cold methanol for 30 min at −20°C and processed with primary antibodies Poly/Mono-ADP Ribose (Cell Signaling Technology, #83 732, 1:1,000 dilution) and anti-PARP1 (Sigma, clone 3G4, WH0000142M1, 1:1,000 dilution) followed by secondaries goat anti-rabbit AlexaFluor 488 and goat anti-mouse AlexaFluor 594 antibodies (Invitrogen, #A-11008, # A-11032, 1:2,000 each); nuclei were counterstained with DAPI (Thermo Fisher Scientific, #D1306, diluted 1 μg/mL in PBS). The nuclear PAR fluorescence intensity in PARP1 positive cells was acquired and analysed using CellInsight CX5 HCS Platform (Thermo Fisher Scientific).

Assessment of the effects of PARG inhibition on PAR levels was performed as follow. SUM149PT or DLD-1 cells were plated at a density of 30 000 cells per well into 96-well plates (Revvity, #6 055 302). PARGi (PDD 00017273, Tocris Bioscience, #5952) was dispensed by an automated digital D300 HP dispenser (Tecan) in titration dilutions, each concentration was tested in duplicate. After 1 h incubation with PARGi, cells were then fixed in ice-cold methanol for 30 min at −20°C and processed for PAR immunostaining as mentioned earlier, except that the anti-PAR antibody (Trevigen, # 4335-MC-100, 1:1000 dilution) was used for the PAR immunofluorescence on DLD-1 cells.

PARylation inhibition with transfected SUM149PT *PARP1* KO was performed as follow. SUM149PT *PARP1* KO cells were plated into 96-well plates (Revvity, #6 055 302). Next day, cells were transfected with pPARP1 (WT or L777P) -IRES-H2B-GFP vectors using the ViaFect Transfection Reagent (Promega, #E4981) and following the manufacturer’s instructions. Twenty-four hours post-transfection, media was changed. Then, PARPi were dispensed by an automated digital D300 HP dispenser (Tecan) in titration dilutions, each concentration was tested in duplicate. Plates were incubated overnight at 37°C, 5% CO_2_. Then, H_2_O_2_ (Camlab Chemicals, #1 110 471) was added at final concentration of 10 mM and cells were incubated for 5 min in culture conditions. Cells were then fixed in ice-cold methanol for 30 min at -20°C and processed with primary antibodies Poly/Mono-ADP Ribose (Cell Signaling Technology, #83 732, 1:1,000 dilution) and anti-PARP1 (Sigma, clone 3G4, WH0000142M1, 1:1,000 dilution) followed by secondaries goat anti-rabbit AlexaFluor 488 and goat anti-mouse AlexaFluor 594 antibodies (Invitrogen, #A-11008, # A-11032, 1:2000 each); nuclei were counterstained with DAPI (Thermo Fisher Scientific, #D1306, diluted 1 μg/mL in PBS). The nuclear PAR fluorescence intensity in PARP1 positive cells was acquired and analysed using CellInsight CX5 HCS Platform (Thermo Fisher Scientific); dose–response curves were generated with GraphPad Prism software.

### Library preparation and whole exome sequencing

DNA was extracted using the AllPrep DNA/RNA Mini kit (Qiagen #80 204). DNA was quantified using the Qubit Flex Fluorometer (Invitrogen), DNA purity was determined using a NanoDrop Eight (Thermo Scientific) and DNA integrity was measured using a 4200 Tapestation (Agilent). Libraries were prepared using the NEBNext Ultra II DNA Library Prep Kit for Illumina (NEB #E7645) and enriched using SureSelectXT Human All Exon V7 (Agilent #5191–4004) probes. Library sizes and quantification were determined by 4200 Tapestation (Agilent), and libraries were subsequently pooled equimolar. The final pool was loaded onto one lane of an S4 v1.5 flow cell (300 cycles) and paired-end sequencing was performed with reads lengths of 150 base pairs (Illumina; 20 028 312).

### Alignment and UMI consensus

Following the completion of each sequencing run, raw sequencing BCL files were transferred to AWS bucket. Subsequently, the run was processed using Illumina DRAGEN pipeline, software version 4.0 (07.021.645.4.0.3).

The demultiplexing step was executed using DRAGEN bcl-to-fastq conversion program bclConvert. The resulting fastq files were aligned to hg38 reference genome employing the alt-aware DRAGEN mapper called DRAGMAP (version of hash table dated 20 220 214, “sw_version”, ”01.003.044.4.0.3″, ”hash_table_version”: “8”). Unique molecular identifiers (UMIs) for all in-house sequenced data were collapsed to a consensus sequence within the DRAGEN pipeline, with a parameter setting of min-reads = 1. A mean coverage of 126.3X (min: 108.3X, max: 165.0X) was achieved.

### Short variant calling

Variant calling was performed using the DRAGEN small variant caller namely dragen-vc as a component of DRAGEN somatic pipeline. Variants with a variant depth below 4 or a total depth below 50 were discarded.

### Expression and purification of CAT PARP1

PARP1(662–1011) and PARP1(662–1011)[L777P] with N-terminal Avi-GS-His6-GS-TEV-GS tags were expressed in Sf21 insect cells from viruses generated from pFastBac1 plasmids for 48 h. Cells were harvested by centrifugation at 3400 g (15 min) and cell pellets were stored at −80°C. Cells were resuspended in equilibration buffer (50 mM HEPES pH 7.5, 500 mM NaCl, 10% glycerol, 1 mM TCEP) supplemented with 20 mM imidazole, 1x EDTA-free protease inhibitor tablets (Roche) and benzonase to 5 mL per gram cells and stored at −70°C for a freeze-thaw lysis. The lysate was clarified by centrifugation at 16 000 rpm at 4°C for 2 h. Proteins were captured on a 5 mL FF NiNTA column (Cytiva), washed with equilibration buffer supplemented with 20 mM imidazole and eluted in equilibration buffer supplemented with 300 mM imidazole.

Proteins for biophysical studies were further purified by size exclusion (S75) in SEC buffer (10 mM HEPES pH 7.5, 150 mM NaCl, 5% glycerol, 1 mM TCEP) before concentrating, flash freezing in liquid nitrogen and stored at -70°C. Proteins were *in vitro* biotinylated using a kit (Avidity) following the manufacturer’s instructions and purified by size exclusion (S75) in SEC buffer, followed by concentrating to 3 mg/mL, flash freezing in liquid nitrogen and stored at −70°C, resulting in >95% purity as determined by SDS PAGE (Supplementary Fig. S6). Intact mass spectrometry analysis confirmed protein identity, complete biotinylation and showed acetylation of both proteins.

### Hydrogen Deuterium Exchange (HDX) mass spectrometry

HDX experiments were performed on a Synapt G2-Si (Waters, UK) qTOF mass spectrometer coupled with an automated HDX system (Waters, UK). The instrument was calibrated with sodium iodide and run in resolution mode. MS^E^ fragmentation with CID was used with electrospray ionisation and ion mobility disabled. The following instrument settings were used: positive polarity, capillary voltage 2.5 kV, source temperature 80°C, sampling cone 30 V, desolvation temperature 150 C, m/z range 50–2000, and transfer collision energy ramp 18–40 V. Leucine enkephalin (Waters, UK) was used as lockmass.

Stock solutions of protein sample (CAT PARP1 WT or CAT PARP1 L777P) were prepared at a concentration of 10 µM containing either 2% DMSO or 20 µM compound for bound or unbound comparison state respectively. The automated HDX system was used to add 57 μL of buffer L (10 mM HEPES, 150 mM NaCl in deuterium oxide and either 2% DMSO or 20 µM compound) to initiate the labelling reaction. The sample was incubated for 0.5, 5, or 30 min in triplicate. For peptide mapping references a 3 μL sample of protein stock was added to 57 μL buffer E (10 mM HEPES, 150 mM NaCl).

ProteinLynx Global Server 3.0.3 (Waters, UK) was used for the peptide map. Peptides were filtered and curated in DynamX 3.0 (Waters, UK). Statistical analysis was performed using Deuteros (King’s College London, UK) using a global confidence interval of 99.9%. Peptides were not corrected for back-exchange. 50 μL of the reaction mixture was quenched with 50 μL of quench buffer (100 mM potassium phosphate pH 2.3) at 4°C. 95 μL of the quenched mixture was injected onto the HDX system. All samples were digested online through a Waters Enzymate BEH pepsin column at 20°C and trapped on a Waters BEH C18 VanGuard column at 0°C. Digestion and trapping were carried out at 100 μL/min for 3 min with 100% mobile phase A (0.1% formic acid in water). Peptides were separated on a Waters BEH C18 column (50 mm x 1.0 mm) at 0°C over a 10 min gradient of 10–30 % of mobile phase B (0.1% formic acid in acetonitrile) at 80 µL/min followed by 2 min of a sawtooth gradient to minimise carryover. A clean blank sample of 0.1% formic acid was injected between every sample for carryover assessment. The pepsin column was washed after every injection with pepsin wash solution (1.5 M Guanidine HCl, 4% acetonitrile, 0.8% formic acid). Statistics and peptides lists results are contained in Supplementary Table S1.

### *In vitro* PARylation assay

Recombinant CAT PARP1 WT or CAT PARP1 L777P were diluted at 100 nM in buffer A (20 mM Tris pH 7.5, 50 mM NaCl, 5 mM MgCl_2_, 0.02% Triton X-100) in a 20 µL reaction. Then, 2 µL of 1 mM NAD (Sigma-Aldrich, #481 911) were added, and reactions were incubated at 37°C for 20 min. Reactions were stopped with the addition of 6 µL of LDS buffer (Themo Fisher Scientific, #NP0007) and analysed by western blot, as detailed earlier.

### Surface plasmon resonance (SPR) analysis

Experiments were performed using Biacore 8K(+) instruments (Cytiva) using a running buffer containing 20 mM HEPES pH 7.4, 150 mM NaCl, 50 μM EDTA, 0.1 mM TCEP, 1% (v/v) DMSO and 0.05% (w/v) Tween-20. All binding experiments were performed with the flow cell temperature set to 25°C, and a flow rate of 30 μL/min was used. The sample chamber was held at 20°C throughout the experiments.

Due to the slow dissociation kinetics of the analytes, a regenerable immobilisation strategy was employed [36]. A biotinylated sensorchip (BHC200M, XanTec bioanalytics) was used to capture coincubated solutions of biotinylated Avi-tagged target (PARP1 WT and PARP1 [L777P]) and SwitchAvidin (SwA)(120 s contact time, 2 μL/min) [37]. Analytes were then tested using single-cycle kinetic titrations (0.00781, 0.0156, 0.0313, 0.0625, 0.125, 0.25, 0.5, 1, 2 μM) with 60 s per concentration followed by a final dissociation time of 3600 s. All bound material was stripped from the biotinylated surface using a solution of 0.25% (w/w) SDS/2.5% (w/w) citric acid in preparation for the capture of fresh target/SwA and testing of further compounds. Sensorgrams for blank subtractions were collected in an identical manner, except the titration solutions contained only running buffer. For analysis of the single-cycle kinetics data, solvent-corrected, reference and blank-subtracted sensorgrams were fit to a 1:1 binding model (with a drift component or bulk refractive shift where required) using Biacore Insight Evaluation 4.0 (Cytiva).

As saruparib exhibited very slow dissociation kinetics, a chaser experiment was performed using a compound known to be competitive with saruparib (but with rapid dissociation kinetics), to more accurately determine the dissociation rate constant for saruparib [38]. Briefly, the chaser compound was injected prior to the saruparib single-cycle kinetic titration (pre-chaser), and at multiple timepoints afterwards (the chasers) at 1 μM (30 s contact time, 180 s dissociation) to monitor the fractional occupancy of the immobilised target with saruparib. A control experiment was performed in parallel, where no test compound was injected during the single-cycle kinetics step, to allow adjustment for surface activity loss during the dissociation period. The surface activity adjusted chaser responses were then normalised to the pre-chaser and then subtracted from 1 to give the fractional occupancy of saruparib as a function of time. These data were then fit in GraphPad PRISM to a single exponential equation to yield the dissociation rate constant for saruparib.

### Complementation of *PARP1* KO cells and cell survival assay

SUM149PT *PARP1* KO cells were first transfected with the indicated plasmids using the ViaFect Transfection Reagent (Promega, #E4981) and following the manufacturer’s instructions. Twenty-four hours post-transfection, transfected cells were seeded onto 96-well plates (Revvity, #6 055 302) at a density of 1000 cells per well. PARPi were dispensed by an automated digital D300 HP dispenser (Tecan) in titration dilutions, each concentration was tested in triplicate and DMSO was used as untreated control. Plates were incubated at 37°C, 5% CO_2_ for 7 days. Cells were then fixed in ice-cold methanol for 30 min at −20°C, washed with PBS then nuclei were counterstained with DAPI (Thermo Fisher Scientific, #D1306, diluted 1 μg/mL in PBS). Nuclei counts per well were quantified using CellInsight CX5 HCS Platform (Thermo Fisher Scientific); dose–response curves were generated with GraphPad Prism software.

### Statistical analysis

The number of independent experiments is indicated in the figure legends. Unless otherwise stated, data are shown as the mean ± S.D. Analysis was performed in Prism 8 (GraphPad Software). *P < 0.05; **: *P*< 0.01; ***: *P*< 0.001, ^****^: *P* < 0.0001.

## Results

### Efficacy of PARPi in *BRCA1*m cells is PARP1-dependent and correlates with PARP1 trapping

**Figure 1.**
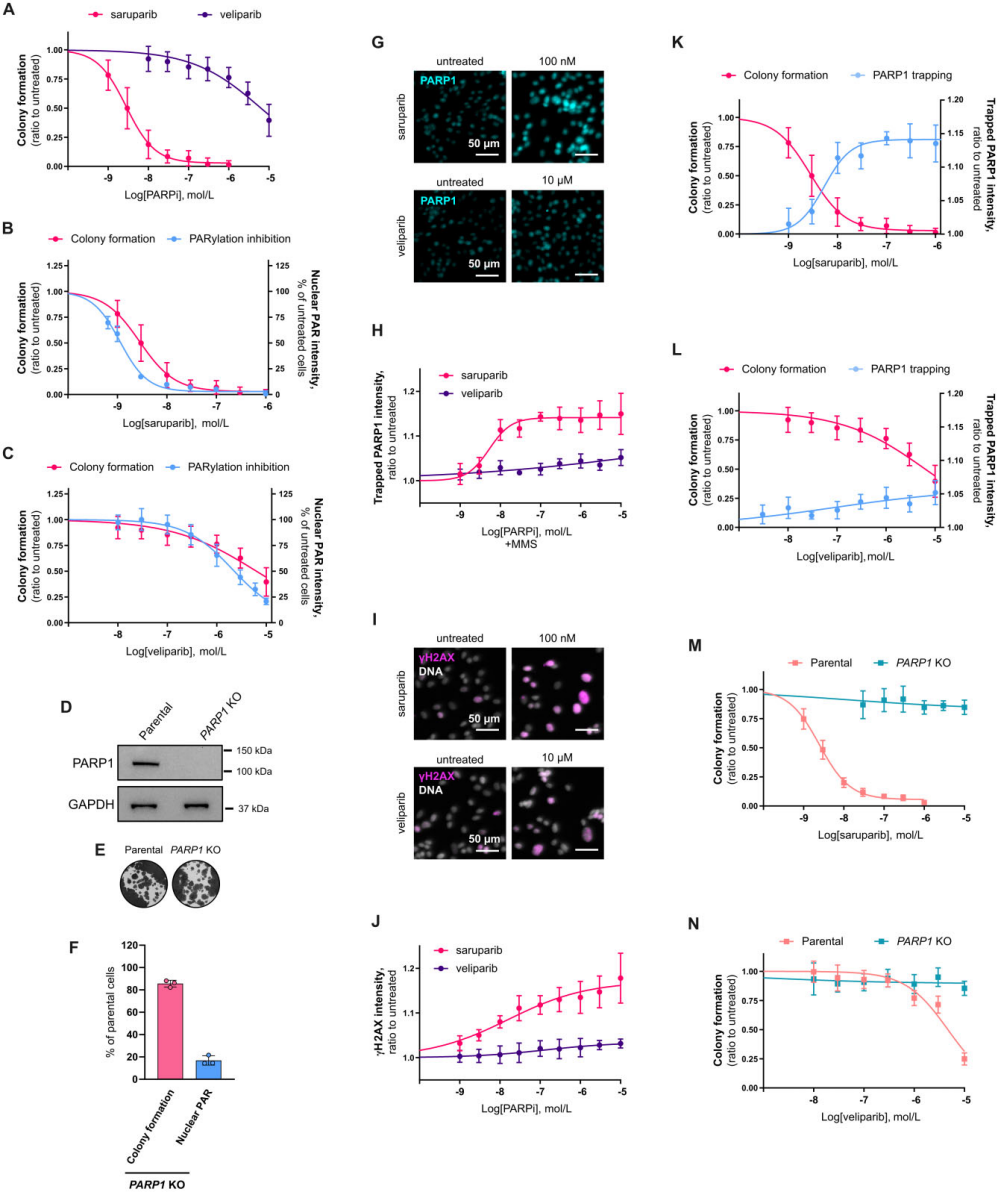
PARP1-dependent efficacy of PARPi in *BRCA1*m cells and correlation with PARP1 trapping. (**A**) Quantification by colony formation assay of the survival of parental SUM149PT cells after 9 days exposure to saruparib or veliparib. (**B** and **C**) Cell survival by colony formation assay and PARylation inhibition dose-responses for saruparib (**B**) or veliparib (**C**). (**D**) PARP1 protein expression levels in parental and *PARP1* KO SUM149PT cells. GAPDH is used as loading control. (**E**) Representative images of a colony formation assay with parental and *PARP1* KO SUM149PT cells. (**F**) Effect of PARP1 loss in SUM149PT cells on colony formation capacity and on PARylation levels after exposure to DNA damaging agent H_2_O_2_ (10 mM). (**G**) Representative immunofluorescence images of PARP1 trapping in SUM149PT cells treated with saruparib or veliparib and the DNA damaging agent MMS (0.005%). (**H**) Quantification of the PARP1 trapping immunofluorescence shown in (**G**). (**I**) Representative immunofluorescence images of γH2AX in SUM149PT cells after 3 days exposure to saruparib or veliparib. (**J**) Quantification of the γH2AX immunofluorescence shown in (**I**). (**K**, and **L**) Cell survival by colony formation assay and PARP1 trapping dose-responses for saruparib (**K**) or veliparib (**L**). (**M** and **N**) Cell survival by colony formation assay of parental or *PARP1* KO SUM149PT cells treated for 9 days with saruparib (**M**) or veliparib (**N**). In all plots, data shown are the means of at least three independent experiments, and error bars indicate ± SD. *P* values calculated using an unpaired t-test.

The SUM149PT breast cancer cell line was chosen for this study as a relevant model for studying PARPi activity, since it harbours a hypomorph *BRCA1* allele with a mutation in the exon 11 of the gene (2288delT causing partial exon 11 skipping (Δ11q)) [22, 39–41]. Given that the PARP1-selective inhibitor, saruparib, exhibits a potent effect on SUM149PT survival (half-maximal inhibitory concentration, or IC_50_ value of 2.8 nM), while the non-selective PARP1/PARP2 inhibitor, veliparib, shows significantly weaker effects (IC_50_ value of 6.3 µM) (Fig. 1A and Supplementary Fig. S1A), we selected these two PARPi as tool compounds to investigate the properties of PARPi that contribute to their efficacy in *BRCA1*m cancer cells. We observed a strong correlation between PARylation inhibition and cell survival dose-responses for saruparib and veliparib, where saruparib, being the most potent enzymatic inhibitor, also showed greatest efficacy (Fig. 1B and C, Supplementary Fig. S1B and C). In accordance with a previous report [27], we observed that PARP1 is not essential for the survival of SUM149PT cells (Fig. 1D and E). PARP1 deficiency only leads to a moderate loss of survival in SUM149PT cells, despite greatly reduced nuclear PAR levels (Fig. 1F and Supplementary Fig. S1D), results that argue PARPi activity is more than just regulation of PAR levels when it comes to cell cytotoxicity (Supplementary Fig. S1E). Collectively, our results suggest that inhibition of the PARylation activity of PARP1 may be necessary but is not sufficient to drive efficacy of PARPi in *BRCA1*m SUM149PT cells.

We next assessed the contribution of PARP1 trapping towards PARPi activity in *BRCA1*m cancer cells. Exposure to saruparib resulted in potent induction of PARP1 trapping and DNA damage, measured by phosphorylation of histone variant H2AX on Ser-139 (γH2AX), while the veliparib effects were modest (Fig. 1G–J). Consistently, we also observed a strong correlation between PARP1 trapping and cell cytotoxicity dose-responses, for both saruparib and veliparib (Fig. 1K and L and Supplementary Fig. S1F).

These results prompted us to investigate whether loss of PARP1 could rescue the sensitivity of SUM149PT cells to saruparib and veliparib, as described previously for other PARPi [27]. In line with previous reports, the absence of PARP1 completely abolished the effect of saruparib and veliparib on the survival of SUM149PT cells (Fig. 1M and N, Supplementary Fig. S1G and H). Collectively, these results suggest that the presence of PARP1 is essential for the efficacy of PARPi in *BRCA1*m SUM149PT cells, a conclusion echoed by numerous studies in the literature [8, 9, 27].

### PARPi selectively induce cytotoxic PARP1 trapping in HRR-deficient cells

**Figure 2.**
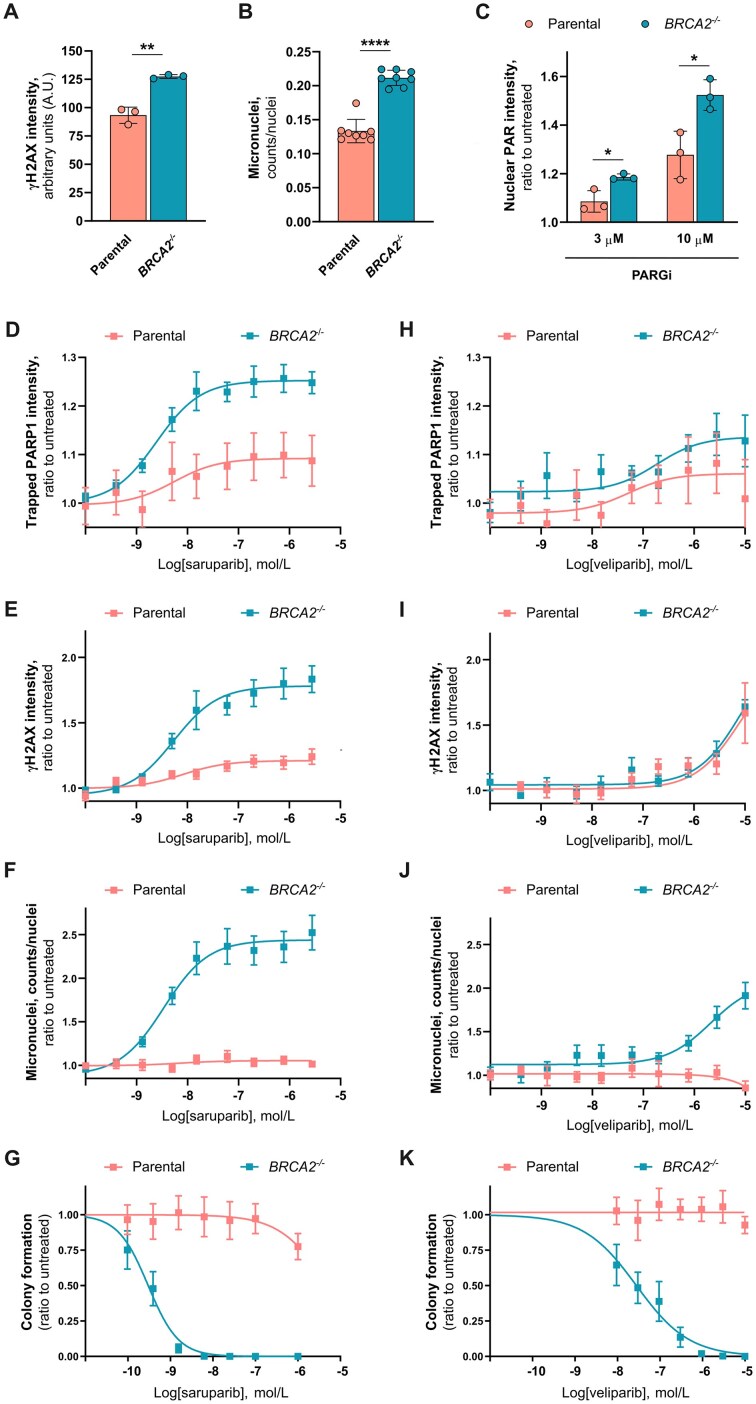
Selective induction of PARP1 trapping by PARPi in HRR-deficient cells and in absence of exogenous DNA damage. (**A**) Quantifications of immunofluorescence analysis of the DNA damage marker γH2AX in untreated parental and *BRCA2^-/-^* DLD-1 cells. (**B**) Micronuclei quantification in untreated parental and *BRCA2*^-/-^ DLD-1 cells. (**C**) Quantifications of immunofluorescence analysis of PAR in parental and *BRCA2^-/-^* DLD-1 cells, after 1 h exposure to a PARG inhibitor (PARGi, PDD 00017273). (D,E) Quantifications of immunofluorescence analysis of PARP1 trapping (**D**) and γH2AX (**E**) in parental and *BRCA2^-/-^* cells, after 72 h treatment with saruparib. (**F**) Micronuclei quantification in parental and *BRCA2^-/-^* cells, after 72 h treatment with saruparib. (**G**) Colony formation assays with DLD-1 isogenic cell line pair, parental and *BRCA2^-/-^*, treated with saruparib. (H,I) Quantifications of immunofluorescence analysis of PARP1 trapping (**H**) and γH2AX (**I**) in parental and *BRCA2^-/-^* cells, after 72 h treatment with veliparib. (**J**) Micronuclei quantification in parental and *BRCA2^-/-^* cells, after 72 h treatment with veliparib. (**K**) Colony formation assay with DLD-1 isogenic cell line pair, parental and *BRCA2^-/-^*, treated with veliparib in dose–response. Data shown in plots are the means of at least three independent experiments. For panels (**A–C**), (**G**), and (**K**), error bars indicate ± SD. For panels (**D–F**) and (**H–J**), error bars indicate ± SEM. *P*-values calculated using an unpaired t-test.

Our observations support the importance of PARP1 trapping for the cytotoxicity of PARPi in HRR-deficient cells. First, we investigated whether HRR-deficient cells spontaneously display higher levels of DNA damage, necessary to recruit and activate PARP1. Consistent with their DNA repair deficiency, we observed that untreated *BRCA2^-/-^* cells accumulate higher levels of γH2AX and micronuclei, markers of DNA damage and genomic instability, compared to the parental cells (Fig. 2A and B, Supplementary Fig. S2A and B). We also observed a selective accumulation of PARylation in *BRCA2*^-/-^ cells after short-term inhibition of PARG (PARGi, PDD 00017273) [42], suggesting significantly more active PARP in *BRCA2^-/-^* cells than in HRR-proficient cells (Fig. 2C and Supplementary Fig. S2C). Interestingly, PARylation levels are higher during S-phase after PARG inhibition, in comparison to other phases of the cell cycle (Supplementary Fig. S2D). Furthermore, *BRCA2^-/-^* cells display higher PAR levels than parental cells during S-phase, suggesting that *BRCA2^-/-^* cells encounter higher levels of PARP binding substrates than parental cells during S-phase, as supported by the literature [43, 44].

Taking into account the elevated baseline levels of DNA damage and PARP activation in *BRCA2^-/-^* cells (Fig. 2A–C), we decided to measure PARP1 trapping after exposure to PARPi and in absence of any exogenous sources of DNA damage, aiming to reconstruct the molecular mechanisms that occur in HRR-deficient versus HRR-proficient cancer cells treated with PARPi monotherapy. To do so, we treated DLD1 *BRCA2^-/-^* and wild-type cells for 72 h, a time point at which cell death is not yet observable, as indicated by the lack of increase in sub-G1 populations but the accumulation of cells in S/G2 phases following saruparib treatment (Supplementary Fig. S2E). Of note, we observed that saruparib preferentially induced PARP1 trapping in *BRCA2^-/-^* cells compared to the wild-type cells (Fig. 2D). In parallel, we could also measure a significant accumulation of DNA damage and micronuclei selectively in *BRCA2^-/-^* cells, recapitulating the cytotoxic phenotype of saruparib in these HRR-deficient cells (Fig. 2E–G and Supplementary Fig. S2F). Interestingly, we were also able to detect a moderate induction of PARP1 trapping by saruparib in DLD-1 wild-type cells (Fig. 2D). However, this did not translate into micronuclei generation nor cytotoxicity (Fig. 2F and G), suggesting that the DNA repair capability of the wild-type HRR-proficient cells prevents the genotoxicity induced by PARP1 trapping. Notably, veliparib treatment resulted in modest PARP1 trapping and DNA damage accumulation, with no significant differences between wild-type and *BRCA2^-/-^* cells (Fig. 2H and I). However, micromolar concentrations of veliparib could induce micronuclei formation, but only in *BRCA2*-deficient cells, leading to cytotoxicity and illustrating the importance of HRR deficiency to convert PARPi-induced DNA damage into genomic instability (Fig. 2J and K and Supplementary Fig. S2G). Together, our data indicate that PARPi can induce PARP1 trapping in a physiological setting in HRR-deficient cells, without any source of exogenous DNA damage. This is in line with previous reports describing the elevated presence of PARP1 binding substrates in HRR-deficient cells [43, 44].

### Generation of cellular models exhibiting resistance to saruparib

**Figure 3.**
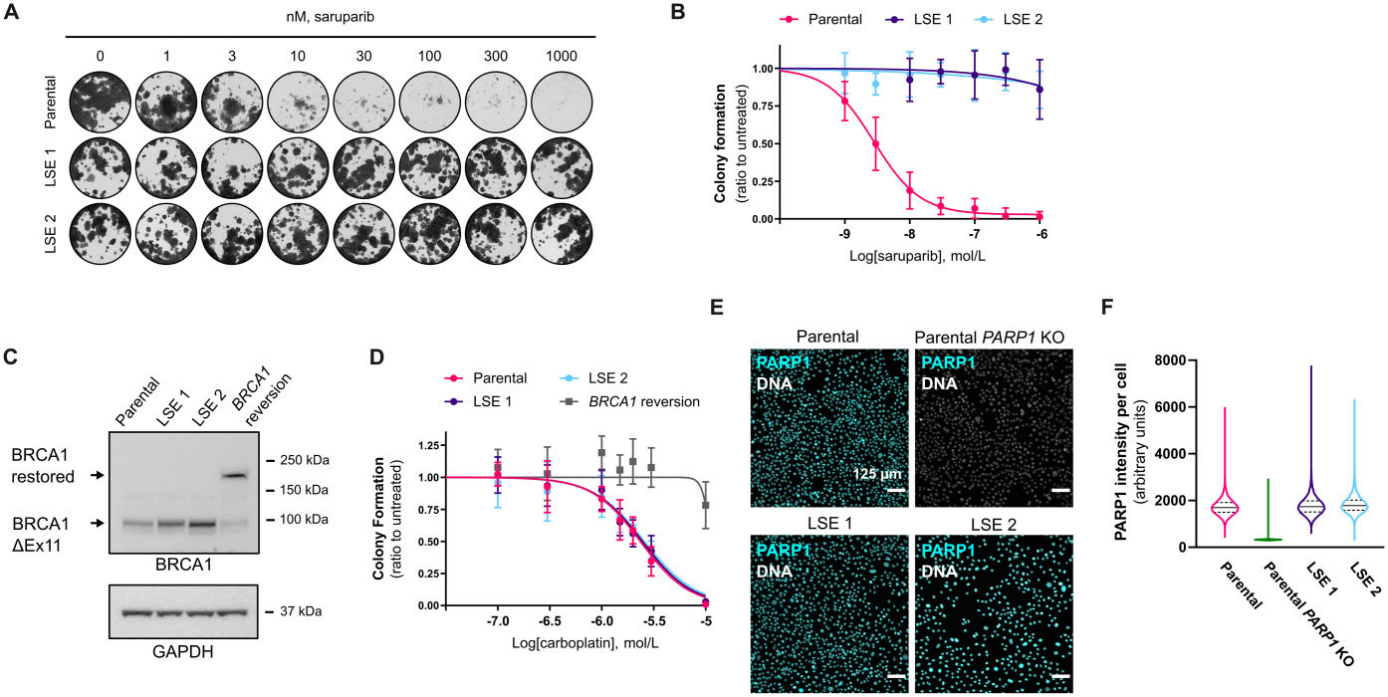
LSE cells do not display *BRCA1* reversion nor loss of PARP1 expression. (**A**) Representative images and (**B**) quantifications of colony formation assays performed with parental SUM149PT cells and the LSE clones 1 and 2 treated with saruparib for 9 days. (**C**) BRCA1 protein expression assessed by western blot in parental, LSE and *BRCA1* reversion SUM149PT cells; GAPDH is used as loading control. Hundred micrograms of proteins were loaded per well. (**D**) Carboplatin sensitivity assessed by colony formation assay after a 9 days exposure. (**E**) PARP1 protein expression assessed by immunofluorescence in parental, LSE and *PARP1* KO SUM149PT cells. (**F**) Quantification of the PARP1 immunofluorescence shown in (**E**); PARP1 expression was assessed in a minimum of 20,000 cells per genotype. Data shown are medians and quartiles. Unless stated otherwise, data shown in plots are the means of at least three independent experiments, and error bars indicate ± SD.

If PARP1 trapping is required for the efficacy of saruparib, PARP1 mutations impairing this activity might be expected upon adaptation to saruparib treatment. To assess this, we cultured SUM149PT cells in the presence of saruparib and generated multiple adapted clones, which we designated as “LSE” for “loss of saruparib efficacy” (Fig. 3A and B).

To elucidate the mechanism behind the observed loss of efficacy of saruparib in our cellular models, we followed up on a previous report that had shown secondary mutations that restored the open reading frame in the *BRCA1* 2288delT allele harboured by SUM149PT cells (termed here “*BRCA1* reversion”) could confer resistance to PARPi [33] (Supplementary Fig. S3A–D). We evaluated the expression of BRCA1 protein in our LSE cells. We could only detect the presence of the parental BRCA1 Δexon 11 hypomorph (Fig. 3C). Consistent with this lack of *BRCA1* gene ORF restoration, the LSE cells did not exhibit increased resistance to carboplatin, unlike cells expressing the *BRCA1* reversion allele (Fig. 3D and Supplementary Fig. S3E). These results indicate that the mechanism responsible for the loss of efficacy of saruparib in our cellular models does not appear to involve restoration of BRCA1 function.

Next, we proceeded to investigate whether the loss of efficacy of saruparib in LSE cells could be due to loss of its target, PARP1. To this end, we evaluated PARP1 expression levels but found little difference between the SUM149PT and LSE clones, suggesting that the diminished efficacy of saruparib in LSE cells is not a consequence of PARP1 protein loss (Fig. 3E and F and Supplementary Fig. S3F).

### Both saruparib-associated PARP1 inhibition and trapping are affected in LSE cells

**Figure 4.**
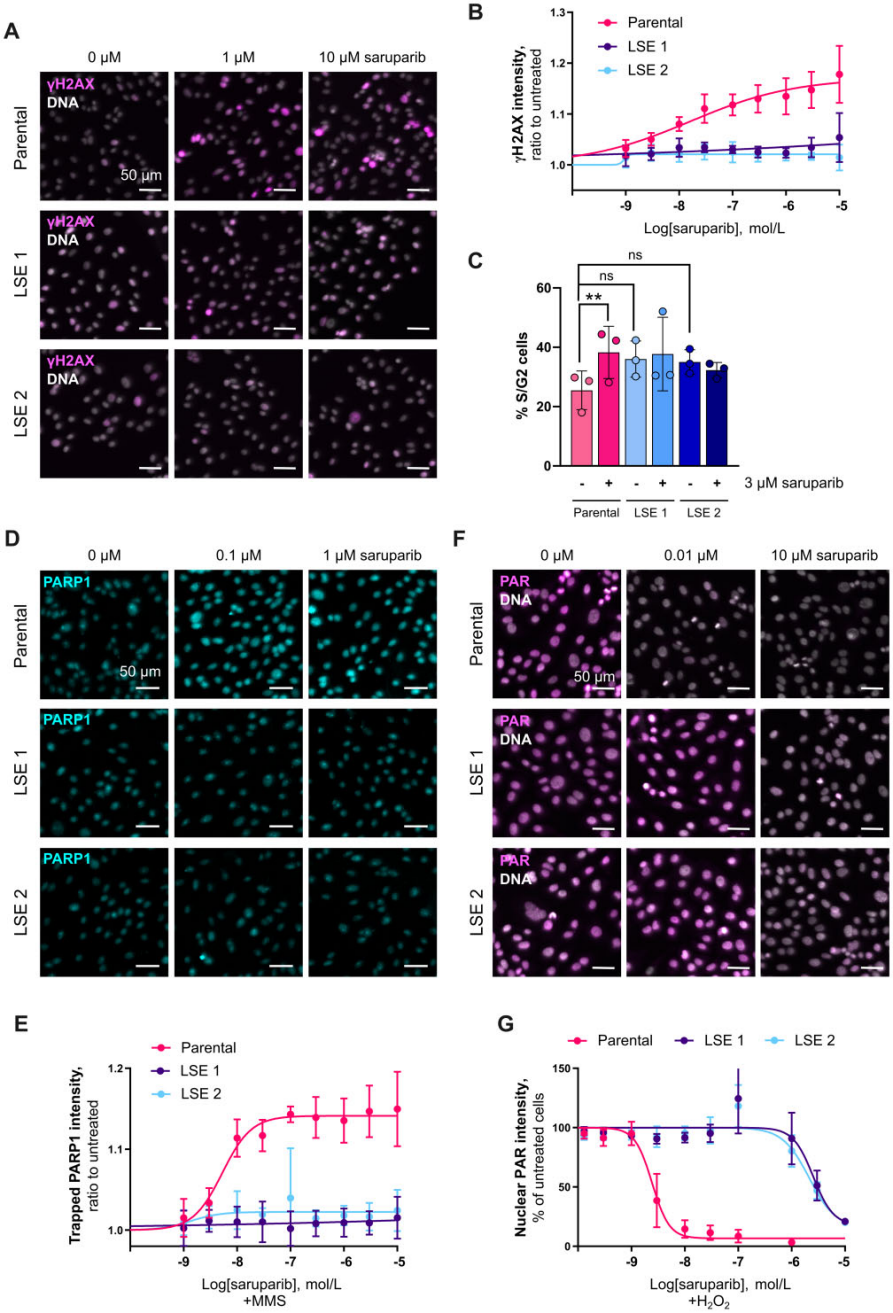
Profiling of the activities of saruparib in LSE cells. (**A**) Representative immunofluorescence images, (**B**) quantifications of immunofluorescence analysis of the DNA damage marker γH2AX and (**C**) cell cycle analysis in parental and LSE SUM149PT cells, after 72 h treatment with saruparib. (**D**) Representative immunofluorescence images of PARP1 trapping in parental and LSE SUM149PT cells treated with saruparib and the DNA damaging agent MMS (0.005%). (**E**) Quantification of the PARP1 trapping immunofluorescence shown in (**D**). (**F** and **G**) PARylation inhibition assay in parental and LSE SUM149PT cells treated with saruparib and the DNA damaging agent H_2_O_2_ (10 mM). In all plots, data shown are the means of at least three independent experiments, and error bars indicate ± SD. *P* values calculated using a ratio paired t-test.

Since neither *BRCA1* reversion nor PARP1 loss could explain the diminished efficacy of saruparib in LSE cells, we sought to characterise the pharmacodynamic markers associated with saruparib treatment [30]. First, we measured the generation of DNA damage following saruparib exposure using γH2AX analysis. Cells were exposed to saruparib for 72 h, to allow SUM149PT cells to undergo at least two cell divisions, considering that the doubling time of these cells is approximately 34 h and PARPi only induce a robust DNA damage response during the second S phase [45–47]. At this timepoint, SUM149PT cells do not undergo cell death and start the DNA damage response (Supplementary Fig. S4). Although saruparib treatment led to a dose-dependent increase in the signal of the DNA damage biomarker γH2AX in parental SUM149PT cells, this was not observed in LSE cells (Fig. 4A and B). Furthermore, cell cycle analysis revealed an accumulation of cells in the S/G2 phase (suggesting DNA damage checkpoint activation) in parental SUM149PT cells treated with saruparib, not observed in LSE cells (Fig. 4C and Supplementary Fig. S4). Together, these findings indicate that saruparib is unable to induce DNA damage in LSE SUM149PT cells, providing a likely explanation for the loss of efficacy in these models.

Next, we sought to assess the PARP1 trapping ability of saruparib in the LSE cells. We observed that saruparib treatment did not result in PARP1 trapping, unlike its effect in parental cells (Fig. 4D and E). This prompted us to investigate whether saruparib retains its ability to inhibit the enzymatic activity of PARP1 in these models. To this end, we evaluated the PARylation inhibition capability of saruparib and observed a dramatic decrease in potency in the LSE cells, with a 1000-fold reduction (Fig. 4F and G). This suggests that, in addition to its impaired trapping activity, saruparib has also lost its ability to effectively inhibit PARP1 in LSE cells.

### LSE cells express a constitutively active PARP1 mutant

To identify the aetiology of the loss of efficacy of saruparib in our cellular models, we performed whole exome sequencing (WES) on both parental and LSE SUM149PT cells and assessed mutations in a manually curated set of genes associated with known mechanisms of resistance to PARPi [48–75]. Of note, a mutation in PARP1 not present in the parental cells, namely L777P (codon change: c.2330T > C, min allele frequency: 0.14, max allele frequency: 0.227), was observed in both LSE clones (Supplementary Fig. S5).

**Figure 5.**
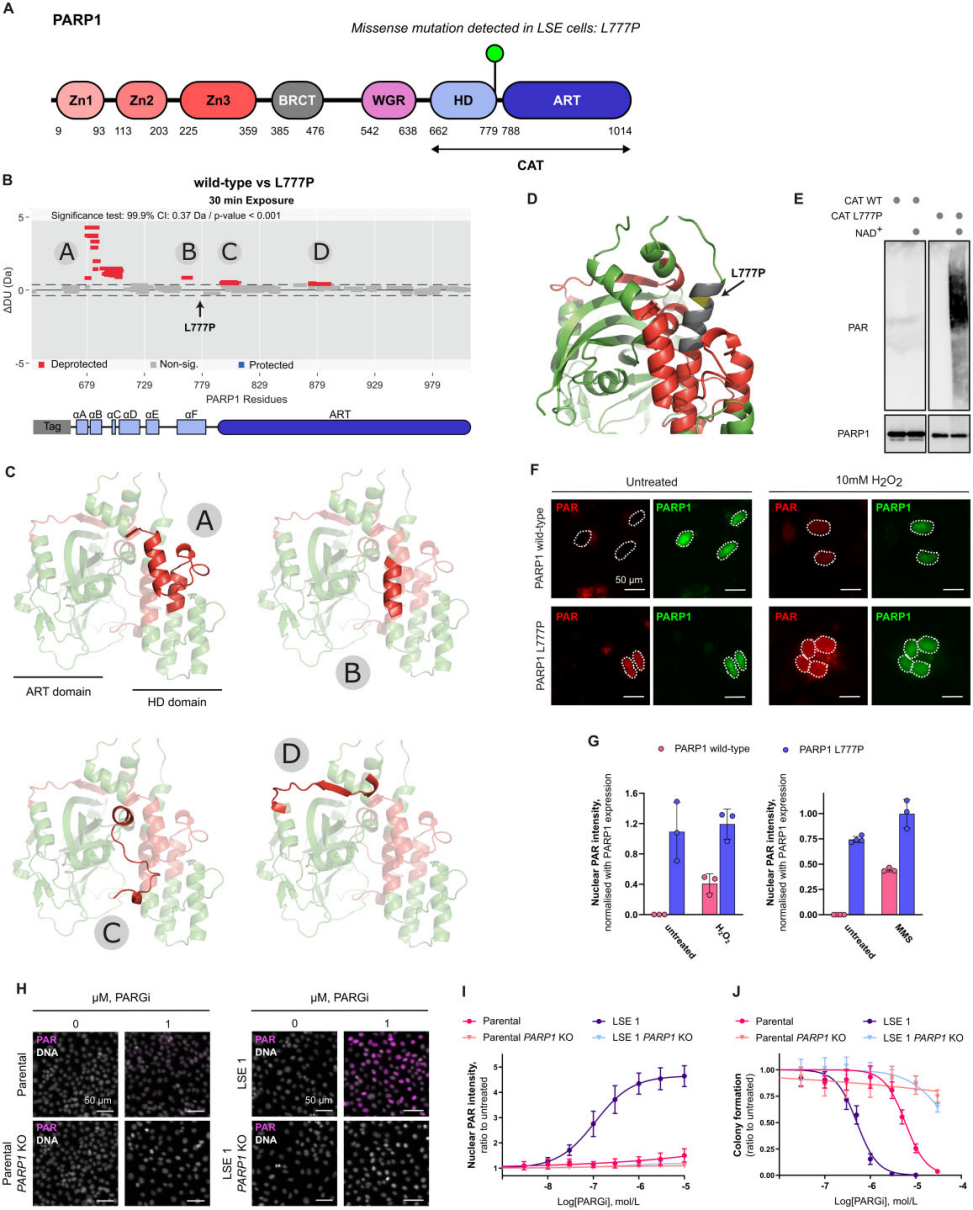
Characterisation of a constitutively active PARP1 mutant expressed in LSE cells. (**A**) Schematic overview of PARP1 protein domains, highlighting the location of the L777P missense mutation identified by WES analysis, and the catalytic domain (CAT) comprised of the HD and ART domains. (**B**) Woods plot from the 30-min timepoint showing the difference in deuterium uptake of peptides in between wild-type and L777P PARP1. A coverage of 88% was achieved across 161 peptides for the comparison of wild-type versus mutant. However, direct comparison at the L777P mutation site was not feasible for differential HDX-MS as peptides with different masses could not be directly compared. Significantly protected or deprotected peptides are shown as blue or red bars, respectively. Non-significant peptides are shown as grey bars. Data are representative of two independent experiments. Regions of interest are called A–D and annotated in panels (**B**) and (**C**). (**C**) Crystal structure of PARP1 CAT domain with regions of significant deprotection highlighted in red (7ONT). (**D**) Zoomed in view of the L777P mutation area. The following areas are displayed in different colours: green for no significance, grey for no coverage, red with significant deprotection, yellow is the mutation L777P. (**E**) Western blot of *in vitro* PARylation assay with wild-type (WT) or mutant L777P recombinant PARP1-CAT proteins, incubated with or without NAD^+^, as indicated. PARP1 is used as protein loading control. (**F**) Representative immunofluorescence images of A549 *PARP1*/*PARP2* KO cells transiently overexpressing wild-type or L777P mutant PARP1, untreated or treated with 10 mM H_2_O_2_. PARP1 expressing nuclei (green) are delineated with dashed circles and PAR signal is displayed in red. (**G**) Quantifications of immunofluorescence analysis shown in (**F**), in untreated cells, and cells treated with 10 mM H_2_O_2_ or 0.01% MMS. (**H**) Representative immunofluorescence images and (**I**) quantifications of immunofluorescence analysis of PAR in parental SUM149PT cells, the clone LSE1 and their respective *PARP1* KOs, after 1 h exposure to a PARG inhibitor (PARGi, PDD 00017273). (**J**) Quantification of colony formation assays performed with parental SUM149PT cells, the clone LSE1 and their respective *PARP1* KOs after 9 days exposure to PARGi. Unless stated otherwise, data shown in plots are the means of at least three independent experiments, error bars indicate ± SD.

The L777P mutation is localised in a structured and regulatory region of PARP1 called the helical domain (HD), which sits inside the catalytic (CAT) domain (Fig. 5A). We sought to investigate the impact of this mutation on the conformational dynamics of PARP1. To this end, we performed differential Hydrogen-Deuterium Exchange Mass Spectrometry (HDX-MS) to analyse both recombinant wild-type and L777P CAT domain of PARP1 (Supplementary Fig. S6). Interestingly, we found a significant deprotection in four distinct regions of the protein sequence as a result of the L777P mutation (regions denoted A–D in Fig. 5B and C, Supplementary Table S1). All identified peptides were in proximity to the L777P mutation, with deprotected peptides in regions A and B specifically located within the HD domain (Fig. 5D). The deprotection in this area suggests increased dynamicity or destabilisation of the HD domain caused by the L777P mutation.

A previous report showed that the enzymatic activation of PARP1 requires local unfolding of its HD domain [11]. Given the results of our structural analysis of PARP1 L777P, we decided to test whether this mutant displayed deregulated PARylation activity. Consistent with findings from previous studies, the isolated CAT region of PARP1 shows very minor PARylation activity due to the absence of the N-terminal region necessary for the DNA-dependent activation of PARP1 (Fig. 5E) [11, 12, 76]. However, we found that the L777P mutant exhibits a greater ability to synthetise PAR in the presence of NAD^+^, suggesting that this mutation produces a constitutively active PARP1 protein (Fig. 5E).

Next, we sought to determine the impact of the L777P mutation on the activity of full-length PARP1 when expressed in cells. To this end, we measured nuclear PAR levels in A549 *PARP1/PARP2* KO cells transiently overexpressing wild-type or mutant PARP1. We observed that cells transiently expressing PARP1 L777P had greater levels of nuclear PAR than those expressing wild-type PARP1 in untreated conditions (Fig. 5F and G, Supplementary Fig. S7A). Furthermore, inducing DNA damage by either hydrogen peroxide or MMS had minimal effects on the PARylation activity of PARP1 L777P (Fig. 5F and G, Supplementary Fig. S7A). Together, these results are consistent with the L777P mutation leading to a constitutively active PARP1 protein.

Since our LSE models harbour the *PARP1* L777P allele, we investigated whether PARP1 exhibited hyperactivity in these cells, under non-stressed conditions. We did not detect any significant difference in nuclear PARylation levels between LSE cells and the parental SUM149PT cells (Supplementary Fig. S7B). This discrepancy may stem from the differences between cells transiently expressing a hyperactive PARP1 variant (A549 cells) and cells metabolically adapted to this PARP1 variant (SUM149PT LSE cells). Considering that long-term accumulation of PAR affects cell survival, we hypothesised that PARG activity might be mitigating the accumulation of nuclear PAR in LSE cells [77, 78]. Therefore, we monitored the accumulation of nuclear PAR after exposure to a PARG inhibitor (PARGi, PDD 00017273) [42]. We observed that PARG inhibition led to a pronounced increase of nuclear PAR signal in LSE cells, in a PARP1-dependent manner (Fig. 5H and I, Supplementary Fig. S7C). Strikingly, LSE cells showed hypersensitivity to PARGi compared to parental SUM149PT cells, and this phenotype was PARP1-dependent (Fig. 5J and Supplementary Fig. S7D). Of note, the LSE cells are not more sensitive than parental SUM149PT cells to the ATR inhibitor, ceralasertib (Supplementary Fig. S7E and F), suggesting that the PARGi hypersensitivity observed in LSE cells is not a result of intrinsically higher levels of replication stress [32, 79, 80]. Additionally, LSE cells exhibited sensitivity to an inhibitor of NAMPT (CHS-828), a key enzyme for NAD^+^ synthesis [81, 82]. In this case, *PARP1* deletion rescued the sensitivity of LSE cells, implying that the increased PARylation activity of the PARP1 L777P mutant significantly depletes NAD^+^ levels in LSE cells (Supplementary Fig. S7G and H). Collectively, these findings indicate that the PARP1 L777P mutation results in constitutive, increased PARylation activity and a corresponding dependency on NAD^+^ synthesis.

### PARP1 L777P mutation transitions saruparib to a PARP1 pro-release inhibitor

Given the dramatic changes in PARylation levels observed in LSE cells (Fig. 5I), which also exhibit loss of PARylation inhibition by saruparib (Fig. 4G), we decided to explore whether the PARP1 L777P mutation could account for this latter phenotype. To investigate this, we evaluated PARylation inhibition by saruparib following exposure to hydrogen peroxide in SUM149PT *PARP1* KO cells transiently overexpressing either wild-type or L777P mutant PARP1. Consistent with results observed in LSE cells, we found that cells expressing PARP1 L777P exhibited a striking 600-fold reduction of the PARylation inhibition activity of saruparib (IC_50_ values of 2.15 nM and 1.34 µM for wild-type and L777P, respectively; Supplementary Fig. S8A and B). Intriguingly, in absence of hydrogen peroxide exposure, saruparib exhibited comparable PARylation inhibition activities in cells expressing wild-type or L777P PARP1 (IC_50_ values of 9 nM and 2 nM for wild-type and L777P, respectively; Supplementary Fig. S8C and D). This finding suggests that saruparib can bind and inhibit PARP1 L777P in the absence of DNA damage. We therefore assessed the affinity of saruparib to PARP1 L777P by Surface Plasmon Resonance (SPR), which indicated very high affinity binding to both wild-type and mutant PARP1 (K_d_ values of 14 and 21 pM for wild-type and L777P, respectively; Supplementary Fig. S8E-K).

**Figure 6.**
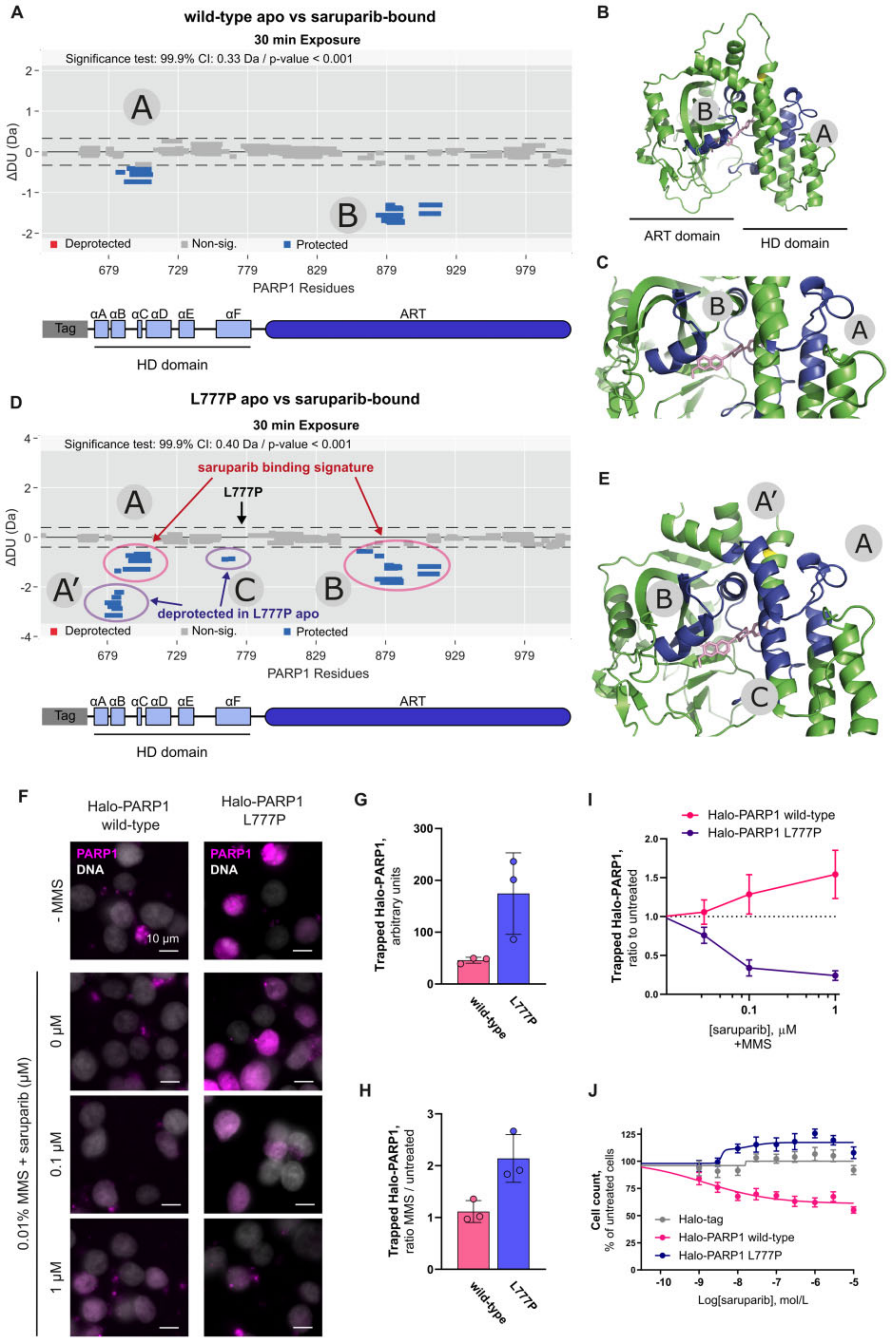
Pro-release activity of saruparib on PARP1 L777P. (**A**) Woods plot for the 30-min timepoint showing the difference in deuterium uptake of peptides in between apo and saruparib-bound wild-type PARP1. A coverage of 90% was achieved across 168 peptides for the comparison between the unbound (apo) and saruparib-bound states of wild-type PARP1. Regions of interest are called A and B and annotated in panels (**B**) and (**C**). (**B**) Crystal structure of PARP1 CAT domain with regions of significant saruparib-induced protection highlighted in blue (7ONT). (**C**) Zoomed in view of binding pocket of saruparib (7ONT). Areas with no significance are displayed in green. Areas with significant protection are displayed in blue. L777 residue is highlighted in yellow. (**D**) Woods plot for the 30-min timepoint showing the difference in deuterium uptake of peptides in between apo and saruparib-bound PARP1 L777P. A coverage of 88% was achieved across 159 peptides for the comparison between the unbound (apo) and saruparib-bound states of the mutant. Regions of interest are called A, A’, C and B and annotated in panel (**E**). Zoomed in view of the binding pocket of saruparib (7ONT). Areas with no significance are displayed in green. Areas with significant protection in (**D**) are displayed in blue. L777P mutation is highlighted in yellow. For wood plots, non-significant peptides are shown as grey bars. Significantly protected or deprotected peptides are shown as blue or red bars respectively. HDX-MS data are representative of two independent experiments. (**F**) Representative images of Halo-PARP1 trapping in HEK293T *PARP1* KO cells transiently overexpressing wild-type or L777P mutant Halo-PARP1, untreated or treated with 0.01% MMS and saruparib. (**G**) Quantification of trapped wild-type or L777P Halo-PARP1 in untreated cells. (**H**) Effect of MMS on the trapping of wild-type or L777P Halo-PARP1 (ratio MMS-treated/untreated cells). (**I**) Effect of saruparib on the trapping of wild-type or L777P Halo-PARP1 (ratio saruparib + MMS-treated/MMS only). Data shown in plots in panels (**G) (H**) and (**I**) are the means of three independent experiments, error bars indicate ± SD. (**J**) Effect of the expression of wild-type or L777P mutant Halo-PARP1 on the survival of SUM149PT *PARP1* KO cells after exposure to saruparib. SUM149PT *PARP1* KO cells transiently overexpressing wild-type or L777P mutant Halo-PARP1 or the Halo-tag were exposed to saruparib for 7 days. Data shown are the means of five independent experiments, error bars indicate ± SEM.

Since the affinity of saruparib for PARP1 is not affected by the L777P mutation, we assessed potential conformational changes of this mutant PARP1 upon binding to saruparib. First, we performed differential HDX-MS to examine the conformational dynamics of wild-type PARP1 upon saruparib binding, revealing increased protection in two distinct regions (Fig. 6A and B, Supplementary Table S1). Region B forms part of the saruparib binding site, as observed by x-ray crystallography with an analogue of saruparib or saruparib [31, 83]. Interestingly, region A does not appear to belong to the binding site of saruparib (Fig. 6C), suggesting that the observed protection in this region might result from long-range effects of saruparib binding.

Next, we proceeded to investigate saruparib effects on the conformational dynamics of the PARP1 L777P mutant. Similar to the observations with the wild-type protein, protection was observed in regions A and B as a result of saruparib binding (Fig. 6D, Supplementary Table S1). Region B forms part of the binding site of saruparib, as observed in the wild-type protein, supporting that saruparib binding is not altered by the L777P mutation (Fig. 6E). Saruparib binding to the L777P mutant also conferred protection in regions outside the established saruparib binding signature (Fig. 6D, Supplementary Table S1). These regions, noted A’ and C, are located within the HD domain of PARP1 and had been previously identified as deprotected in the unbound state of PARP1 L777P (regions A and B in Fig. 5B–D). These findings suggest that regions in the HD domain destabilised by the L777P mutation are re-stabilised upon saruparib binding.

A prior study demonstrated that PARPi that stabilise the HD domain concurrently reduced the affinity of PARP1 for DNA breaks [14]. Given the stabilising effect of saruparib on the HD domain of PARP1 L777P, we asked whether the PARP1 L777P mutation could be causing the loss of trapping activity of saruparib observed in LSE cells (Fig. 4E). To this end, we assessed the trapping activity of saruparib in HEK293T *PARP1* KO cells transiently expressing either Halo-tagged PARP1 wild-type or the L777P mutant (Supplementary Fig. S9A and B). We observed that PARP1 L777P was readily retained on the chromatin even without any treatment, and exposure to MMS further enhanced its chromatin retention (Fig. 6F–H). Note that the apparent cell-to-cell differences in PARP1 L777P trapping signal is to be attributed to the heterogeneous delivery of the PARP1 coding plasmids during the transient transfection of HEK293T *PARP1* KO cells and is not dependent on the cell cycle phase, as the MMS treatment enables the trapping in all cells regardless of their cell cycle phase (Fig. 6F and Supplementary Fig. S9C). Subsequently, we examined the impact of saruparib on the trapping of PARP1 L777P. Unlike its effect on wild-type PARP1, saruparib did not increase the trapping of PARP1 L777P but instead promoted its release from the chromatin (Fig. 6F and I, and Supplementary Fig. S9D).

Of note, in LSE cells PARP1 trapping levels are very similar to the parental cells after MMS exposure (Supplementary Fig. S9E) and saruparib does not alter that PARP1 trapping signal, nor releases PARP1 from the chromatin in LSE cells (Fig. 4E). These phenotypic differences may stem from the fact that LSE cells express both wild-type and L777P *PARP1* alleles (min L777P allele frequency: 0.14, max L777P allele frequency: 0.227, Supplementary Fig. S5). Given that the PARP1 antibody used in our study cannot differentiate the different PARP1 variants, the trapping observed by immunofluorescence in LSE cells are likely a result of both wild-type and L777P variants.

Our findings further prompted us to investigate whether PARP1 L777P expression is causing the loss of saruparib efficacy in the LSE cells. We evaluated the effect of saruparib on the survival of SUM149PT *PARP1* KO cells expressing Halo-tagged PARP1 variants. While reintroducing wild-type PARP1 into SUM149PT *PARP1* KO cells restored their sensitivity to saruparib, the expression of PARP1 L777P did not lead to a decrease in cell survival following saruparib treatment (Fig. 6J). Taken together, these results provide evidence that the PARP1 L777P mutation is indeed responsible for the loss of efficacy of saruparib in LSE cells.

### PARP1 L777P mutation potentiates the trapping activity and efficacy of veliparib

**Figure 7.**
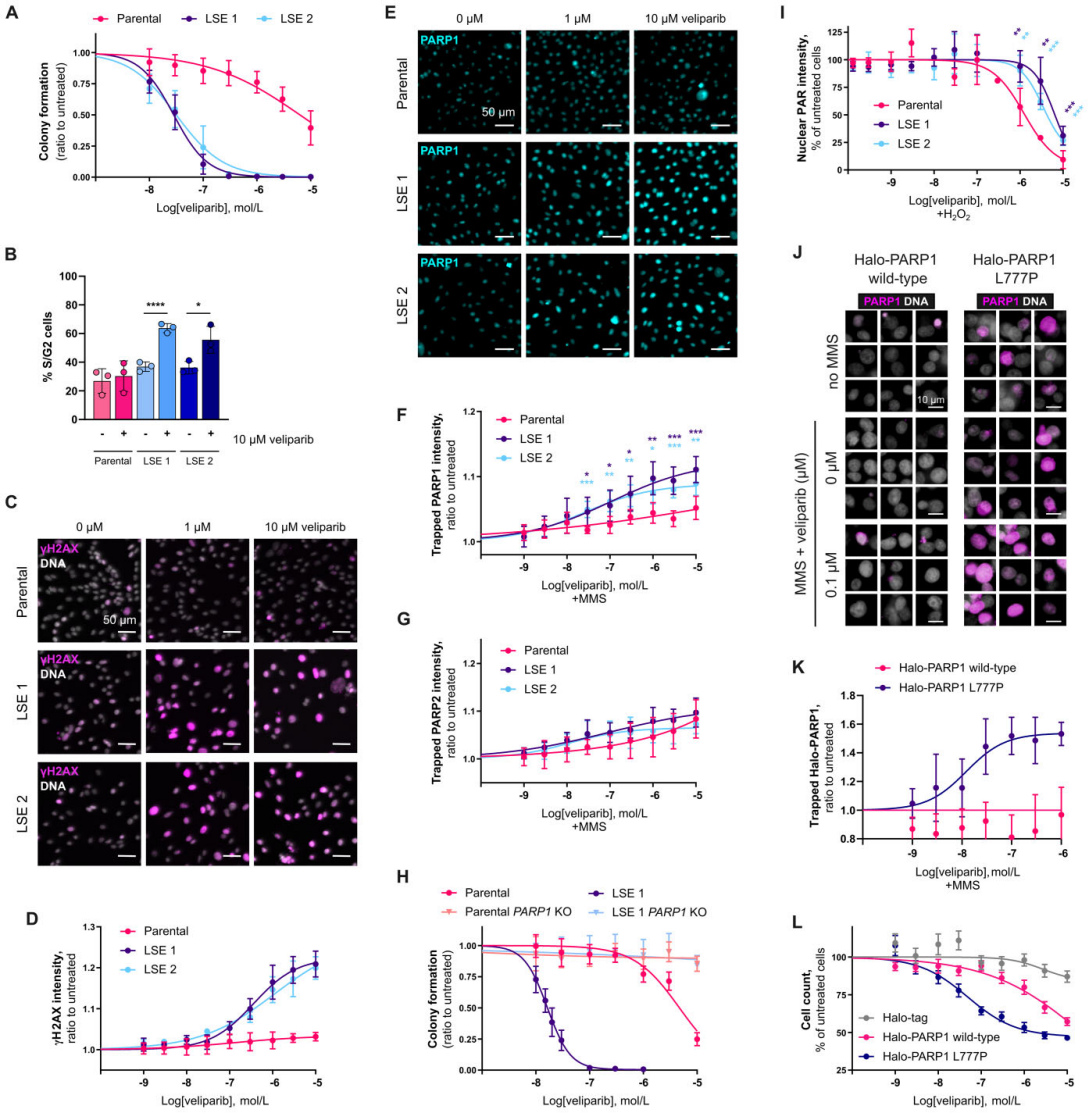
Potentiation of the efficacy and trapping activity of veliparib by the PARP1 L777P mutation. (**A**) Quantification of colony formation assays performed with parental or the LSE SUM149PT cells treated with veliparib for 9 days. (**B**) Cell cycle analysis in parental and LSE SUM149PT cells, after 72 h treatment with veliparib. (**C**) Representative immunofluorescence images and (**D**) quantifications of immunofluorescence analysis of the DNA damage marker γH2AX in parental and LSE SUM149PT cells, after 72 h treatment with veliparib. (**E**) Representative immunofluorescence images and (**F**) quantifications of immunofluorescence analysis of PARP1 trapping in parental and LSE SUM149PT cells, after treatment with veliparib and 0.005% MMS. (**G**) Quantifications of immunofluorescence analysis of PARP2 trapping in parental and LSE SUM149PT cells, after treatment with veliparib and 0.005% MMS. (**H**) Quantification of colony formation assays performed with parental SUM149PT cells, the clone LSE1 and their respective *PARP1* KOs after 9 days exposure to veliparib. (**I**) PARylation inhibition assay in parental and LSE SUM149PT cells treated with veliparib and 10 mM H_2_O_2_. (**J**) Representative images of Halo-PARP1 trapping in HEK293T *PARP1* KO cells transiently overexpressing wild-type or L777P mutant Halo-PARP1, untreated or treated with 0.005% MMS and veliparib. (**K**) Effect of veliparib on the trapping of wild-type or L777P Halo-PARP1 (ratio saruparib + MMS-treated/MMS only). (**L**) Effect of the expression of wild-type or L777P mutant Halo-PARP1 on the survival of SUM149PT *PARP1* KO cells after exposure to veliparib. SUM149PT *PARP1* KO cells transiently overexpressing wild-type or L777P mutant Halo-PARP1 or the Halo-tag were exposed to PARPi for 7 days. Data shown are the means of five independent experiments, error bars indicate ± SEM. Unless stated otherwise, data shown in plots are the means of at least three independent experiments, error bars indicate ± SD. *P*-values calculated using an unpaired t-test.

The effects of the PARP1 L777P mutation on the efficacy of saruparib prompted us to investigate how this mutation influences the efficacy of other PARPi, such as veliparib, a PARPi with weak trapping activity [8]. Surprisingly, we found that LSE cells are two hundred times more sensitive to veliparib than parental cells (IC_50_ of 6.3 µM, 28 nM, and 31 nM for parental, LSE1 and LSE2, respectively) (Fig. 7A and Supplementary Fig. S10A). Exposure to veliparib also resulted in an increased accumulation of S/G2 phase cells in the LSE cells (Fig. 7B and Supplementary Fig. S10B). This phenotype was associated with a significant induction of DNA damage in LSE cells compared to parental cells, as measured with the γH2AX marker (Fig. 7C and D).

Next, we sought to evaluate how the efficacy of veliparib in LSE cells correlates with its PARP trapping ability. Of note, we found that veliparib treatment in the presence of MMS led to higher levels of PARP1 trapping in LSE cells compared to parental cells (Fig. 7E and F), while we could not observe any change in PARP2 trapping (Fig. 7G and Supplementary Fig. S10C). Given these observations, we asked whether the hypersensitivity of LSE cells to veliparib was PARP1-dependent. We found that depletion of PARP1 completely prevented the increased sensitivity to veliparib of LSE cells (Fig. 7H and Supplementary Fig. S10D).

Given the enhanced efficacy of veliparib in LSE cells, we sought to determine if its PARylation inhibition activity was similarly potentiated in these cells. Importantly, we observed that the PARylation inhibition activity of veliparib was, if anything, reduced in LSE cells, compared to parental SUM149PT cells (Fig. 7I and Supplementary Fig. S10E).

The intriguing profile of veliparib activities in LSE cells harbouring the PARP1 L777P mutation led us to investigate whether this mutation is responsible for the potentiation of the PARP1-trapping activity of veliparib. We assessed the trapping activity of veliparib in HEK293T *PARP1* KO cells transiently expressing either wild-type or L777P mutant Halo-PARP1 (Fig. 7J and Supplementary Fig. S10F). We found that in cells expressing PARP1 L777P, veliparib is a potent trapper, whereas, as expected, it exhibits little to no trapping activity with wild-type PARP1 (Fig. 7K). In agreement with results obtained in LSE cells, we also observed that the PARylation inhibition activity of veliparib is reduced in cells expressing PARP1 L777P compared to cells expressing wild-type PARP1 (Supplementary Fig. S10G and H). Finally, we asked how these results translate into the efficacy of veliparib on cell survival. Expression of wild-type PARP1 had a mild effect on veliparib activity in SUM149PT *PARP1* KO cells (Fig. 7L), as expected given the limited efficacy of veliparib in parental SUM149PT cells (Fig. 7H). By contrast, expression of PARP1 L777P hypersensitised cells to veliparib (Fig. 7L). These data strongly suggest that the enhancement of the PARP1 trapping activity of veliparib, without potentiation of the PARylation inhibition activity, ultimately leads to an increase of the cytotoxicity of this inhibitor in *BRCA1*m cancer cells.

### PARP1 L777P mutation separates the PARP1 trapping and PARylation inhibition activities of olaparib and talazoparib

**Figure 8.**
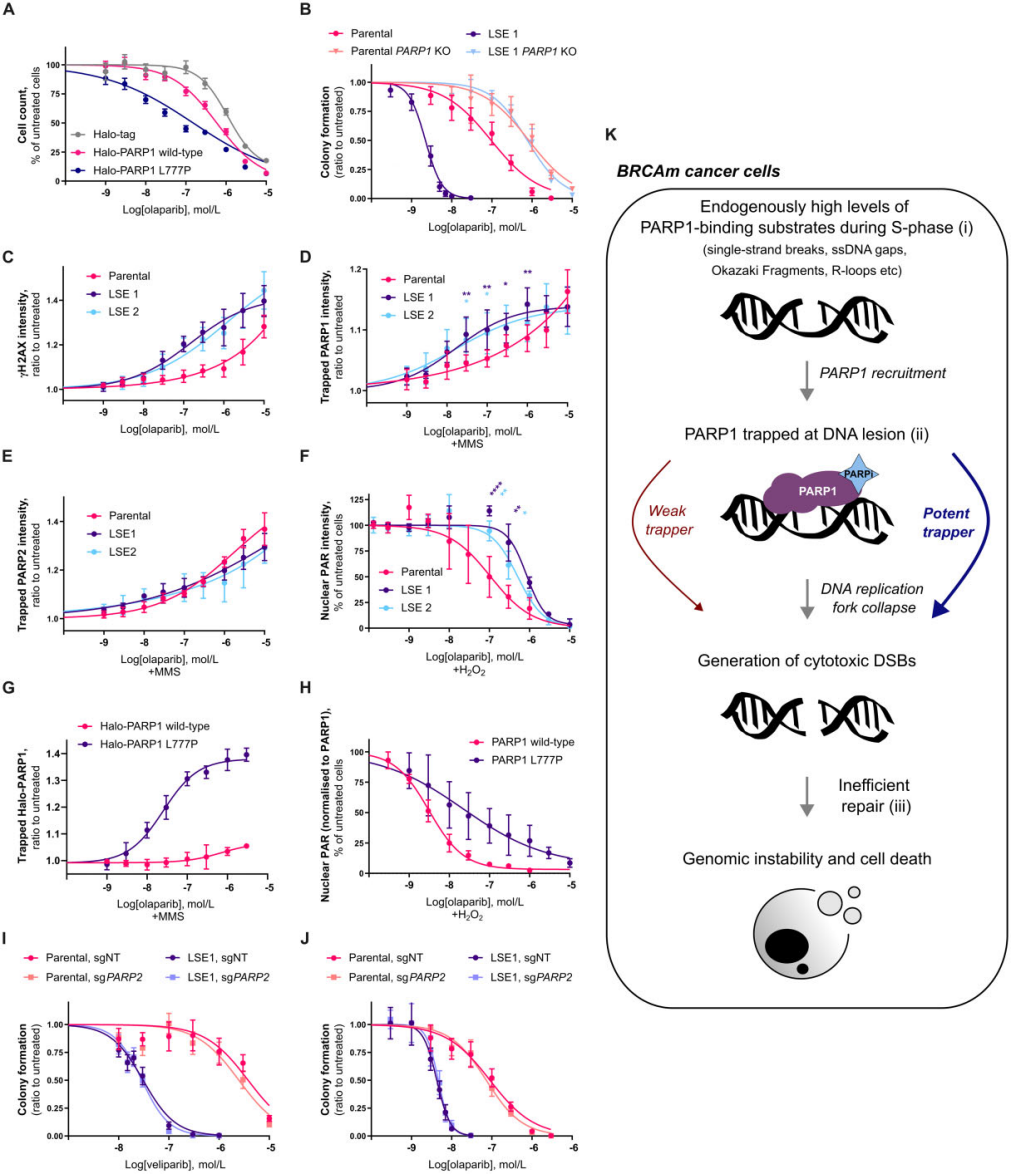
The PARP1 L777P mutation potentiates the efficacy and trapping activity of olaparib, without enhancing the potency of the enzymatic inhibition. (**A**) Effect of the expression of wild-type or L777P mutant Halo-PARP1 on the survival of SUM149PT *PARP1* KO cells after exposure to olaparib. SUM149PT *PARP1* KO cells transiently overexpressing wild-type or L777P mutant Halo-PARP1 or the Halo-tag were exposed to PARPi for 7 days. Data shown are the means of five independent experiments, error bars indicate ± SEM. (**B**) Quantification of colony formation assays performed with parental SUM149PT cells, the clone LSE1 and their respective *PARP1* KOs after 9 days exposure to olaparib. (**C**) Quantifications of immunofluorescence analysis of the DNA damage marker γH2AX in parental and LSE SUM149PT cells, after 72 h treatment with olaparib. (**D**) Quantifications of immunofluorescence analysis of PARP1 trapping in parental and LSE SUM149PT cells, after treatment with olaparib and 0.005% MMS. (**E**) Quantifications of immunofluorescence analysis of PARP2 trapping in parental and LSE SUM149PT cells, after treatment with olaparib and 0.005% MMS. (**F**) PARylation inhibition assay in parental and LSE SUM149PT cells treated with olaparib and 10 mM H_2_O_2_. (**G**) Effect of olaparib on the trapping of wild-type or L777P Halo-PARP1 (ratio saruparib + MMS-treated/MMS only). Data shown are the means of two independent experiments, error bars indicate ± SD. (**H**) PARylation inhibition assay with SUM149PT *PARP1* KO cells transiently overexpressing wild-type PARP1 or PARP1 L777P and treated with olaparib and 10 mM H_2_O_2_. Data shown are the means of two independent experiments, and error bars indicate ± SD. (**I,J**) Quantification of colony formation assays performed with parental SUM149PT cells or the clone LSE1 transfected with a non-targeting sgRNA (sgNT) or with sgRNA targeting *PARP2* (sg*PARP2*), and after 9 days exposure to veliparib (**I**) or olaparib (**J**). Data shown are the means of two independent experiments, and error bars indicate ± SD. Unless stated otherwise, data shown in plots are the means of three independent experiments, error bars indicate ± SD. *P*-values calculated using an unpaired t-test. (**K**) Working model of the mechanism of action of PARP inhibitors in BRCAm cancer cells. Collectively, our data suggest that the selective cytotoxicity of PARPi in BRCAm cells is driven by the combination of (i) the increased presence of PARP1 binding substrates, leading to (ii) increased PARP1 trapping and DNA damage generation, and (iii) defective HRR repair in HRR-deficient genetic backgrounds.

Next, we sought to test if the trapping-dependent increase in veliparib cytotoxicity observed in *BRCA1*m cells expressing the L777P mutant PARP1 can be observed with additional PARPi. We found that expression of PARP1 L777P hypersensitised SUM149PT *PARP1* KO cells to the PARPi olaparib (Fig. 8A). LSE SUM149PT cells also exhibited heightened sensitivity to this PARPi, in a PARP1-dependent manner (Fig. 8B and Supplementary Fig. S11A). As with veliparib, exposure to olaparib led to a significant accumulation of S/G2 phase cells and an induction of DNA damage specifically in the LSE cells (Supplementary Figs S11B, C and Fig. 8C). Of note, we found that olaparib treatment in the presence of MMS induced higher levels of PARP1 trapping in LSE cells compared to parental SUM149PT cells, while we could not observe any change in PARP2 trapping (Fig. 8D and E, Supplementary Fig. S11D and E). However, the PARylation inhibition activity of olaparib was reduced in LSE cells, compared to parental SUM149PT cells (Fig. 8F and Supplementary Fig. S11F), as we observed with veliparib. We also validated that the PARP1 L777P mutation potentiates the PARP1 trapping activity of olaparib (Fig. 8G and Supplementary Fig. S11G), while it reduces the enzymatic inhibition potency of olaparib (Fig. 8H and Supplementary Fig. S11H). Together, our data suggest that the trapping-dependent increase of cytotoxicity, without potentiation of the PARylation inhibition activity, is not exclusive to veliparib but can also be observed with other PARPi, in this case, olaparib.

The fact that the PARP2 trapping activities of veliparib and olaparib are not enhanced in LSE cells suggests that PARP2 may not be involved in the improved efficacy of those PARP1/2i in LSE cells (Figs 7G and 8E). We further tested this by assessing the efficacy of veliparib and olaparib, when *PARP2* is invalidated in LSE cells (Supplementary Fig. S12A). We found that PARP2 loss did not rescue the hypersensitivity of LSE SUM149PT cells to veliparib nor olaparib (Fig. 8I and J, Supplementary Fig. S12B and C). These results suggest that PARP2 is not involved in the hypersensitivity to olaparib and veliparib of LSE cells (Figs 7H and 8B). Of note, PARP2 loss does not re-sensitise LSE cells to saruparib, indicating that PARP2 does not play a role in the loss of efficacy of saruparib in these cells (Supplementary Fig. S12D and E). It is worthwhile noting that *PARP2* invalidation did not reduce the efficacy of neither veliparib nor olaparib in parental SUM149PT cells (Fig. 8I and J, Supplementary Fig. S12B and C). Consequently, PARP2 does not play a role in the efficacy of olaparib and veliparib in *BRCA1*m cancer cells, it is entirely PARP1-dependent.

Next, we profiled the activities of a potent PARP1/2 inhibitor and trapper, namely talazoparib, in SUM149PT cells. We observed that the sensitivity of LSE cells to talazoparib is comparable to the sensitivity of parental cells (Supplementary Fig. S13A and B). Consistently, exposure to talazoparib potently induced DNA damage in both parental and LSE SUM149PT cells (Supplementary Fig. S13C and D). Then, we found that the PARP1 and PARP2 trapping activities of talazoparib remain comparable in both parental and LSE SUM149PT cells (Supplementary Fig. S13E–H). However, the PARylation inhibition activity of talazoparib was reduced in LSE cells, compared to parental SUM149PT cells (Supplementary Fig. S13I and J). Altogether these results align with the notion that the PARP1 trapping activity of PARPi drives their efficacy in *BRCA1*m cancer cells, regardless of their selectivity or PARylation inhibition potency.

### Binding of olaparib or veliparib differentially alter the structure of the HD domain of PARP1 L777P

**Figure 9.**
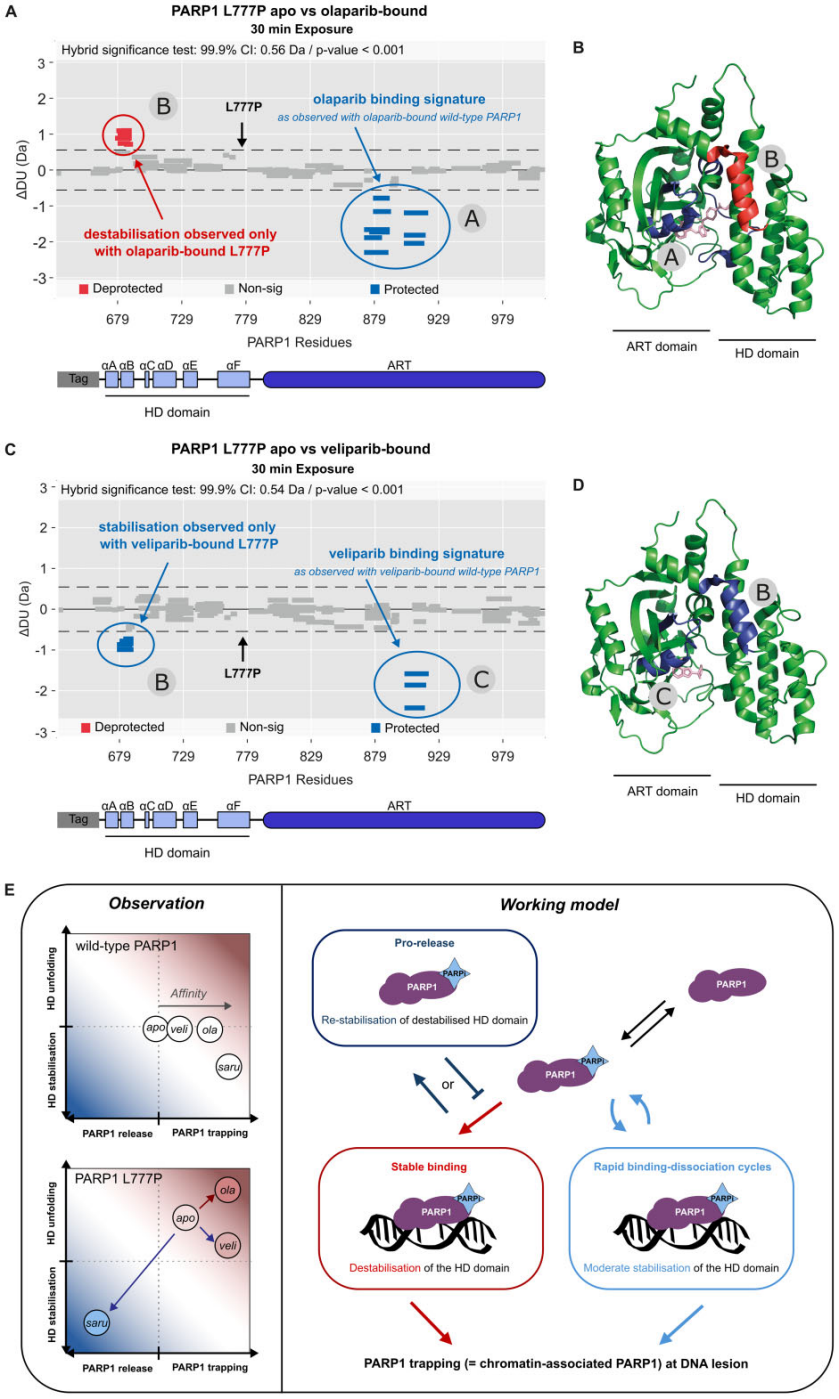
Characterisation of the allosteric effects of olaparib and veliparib on PARP1 L777P. (**A**) Woods plot for the 30-min timepoint showing the difference in deuterium uptake of peptides in between apo and olaparib-bound PARP1 L777P. A coverage of 90.6% was achieved across 146 peptides for the comparison between the unbound (apo) and olaparib-bound states of the mutant. Regions of interest are called A and B and annotated in panel (**B**). (**B**) Crystal structure of PARP1 CAT domain with regions of significant olaparib-induced protection/deprotection highlighted in blue/red (7AAD). Areas with no significance are displayed in green. (**C**) Woods plot for the 30-min timepoint showing the difference in deuterium uptake of peptides in between apo and veliparib-bound PARP1 L777P. A coverage of 90.60% was achieved across 146 peptides for the comparison between the unbound (apo) and veliparib-bound states of the mutant. Regions of interest are called B and C and annotated in panel (**D**). (**D**) Crystal structure of PARP1 CAT domain with regions of significant veliparib-induced protection highlighted in blue (7AAC). Areas with no significance are displayed in green. For wood plots, non-significant peptides are shown as grey bars. Significantly protected or deprotected peptides are shown as blue or red bars respectively. HDX-MS data are representative of two independent experiments. (**E**) Left panel: summary of the observed allosteric changes induced by PARP inhibitors in wild-type and mutant PARP1, along with their respective trapping activities. Right panel: working model illustrating the distinct mechanisms underlying PARP1 trapping and the associated structural changes.

We next sought to determine whether the potent trapping activities of olaparib and veliparib on the PARP1 L777P mutant could stem from potential conformational changes in this mutant upon binding to those PARPi. First, we performed differential HDX-MS to examine the conformational dynamics of wild-type and mutant PARP1 L777P upon olaparib binding. This analysis revealed increased protection in a region of the ART domain (noted A) in both wild-type and mutant PARP1 (Fig.9A and Supplementary Fig. S14A). This region A is part of the olaparib binding site, as confirmed by X-ray crystallography (Fig. 9B and Supplementary Fig. S14B) [84]. Interestingly, olaparib binding to the L777P mutant additionally induced deprotection in its HD domain (noted B), a change not observed in wild-type PARP1 bound to olaparib (Fig. 9A and B and Supplementary Fig. S14A and B). These findings suggest that the binding of olaparib further exacerbates the destabilisation of the HD domain caused by the L777P mutation on PARP1 (Fig. 5B–D).

Finally, we investigated the effects of veliparib on the conformational dynamics of the PARP1 L777P mutant. Veliparib binding resulted in increased protection in a region of the ART domain (noted C) in both wild-type and mutant PARP1 (Fig. 9C and Supplementary Fig. S14C), as observed for olaparib. This region C is part of the veliparib binding site, as confirmed by X-ray crystallography (Fig. 9D and Supplementary Fig. S14D) [84]. Of note, we also found that veliparib binding to the L777P mutant induced protection in its HD domain, in the same region B destabilised by olaparib, a change not observed in the wild-type protein bound to veliparib (Fig. 9C and D and Supplementary Fig. S14C and D). These results suggest that the improved trapping activities of olaparib and veliparib on PARP1 L777P may stem from distinct structural changes in the HD domain.

## Discussion

More than forty years after the discovery of the first PARPi and a decade after the first clinical approval of a PARPi [7, 85, 86], the mechanism of action and the significance of the ability of PARPi to inhibit and trap PARP proteins onto DNA is still a topic of debate [29, 87]. On the one hand, inhibition of the PARylation activity of PARP1 impacts on DNA repair while on the other hand, PARP1 trapping both contributes to the inhibition of DNA repair and creates DNA lesions resulting in increased cytotoxicity in HRR-deficient cancers [26]. Both mechanisms are proposed to be key components of the cytotoxic effects of PARPi single agent activity.

The genetic interaction between *PARP1* and the HRR pathway strongly supports that PARylation inhibition could play an important role in the efficacy of PARPi against HRR-deficient cancers [23, 24]. However, PARylation inhibition and PARP trapping are not mutually exclusive mechanisms. Indeed, to our knowledge, there are no examples of PARPi exhibiting potent trapping activity but no PARylation inhibition ability, suggesting that catalytic inhibition and trapping of PARP1 are linked processes, and that trapping may also contribute to the efficacy of PARPi in HRR-deficient cancer cells [26]. Furthermore, a significant subset of HRR-deficient cancers harbour potential hypomorphic alleles of HRR genes, rather than complete loss-of-function mutations [18–22]. These cancers might be less reliant on the enzymatic activity of PARP1, yet still respond to PARPi treatment at clinically relevant doses (Fig. 1A). Here, we used SUM149PT *BRCA1m* cells as a model for such cancers and confirmed that PARP1 is dispensable for the survival of these cells (Fig. 1D and E) [27]. Therefore, while PARP1 is essential for the survival of cells characterised by complete loss of function of *BRCA1/2* or other HRR genes [23, 24, 29], the residual BRCA1 activity of a BRCA1 hypomorph appears to enable cell survival upon loss of PARP1 enzymatic activity (Fig. 1D and E), as previously published [27]. Nevertheless, we observed that PARP1 presence is necessary for the efficacy of PARPi in these cells, suggesting that PARP1 trapping might drive the efficacy of PARPi in *BRCA1*m cells (Fig. 1M and N).

In our subsequent experiments, we aimed to rigorously test the PARP1 trapping hypothesis and, by generating new cellular models that exhibited loss of efficacy of saruparib, we obtained cells harbouring an acquired point mutation in *PARP1*. We showed that this mutation was the sole cause of the loss of saruparib efficacy in these models, a finding that underpins the high selectivity of saruparib for PARP1, corroborating previous observations [30, 31] (Fig. 6J). Mechanistically, we discovered that the L777P mutation in PARP1 impairs both the PARP1 trapping and PARylation inhibition activities of saruparib (Fig. 6I and Supplementary Fig. S8A). Thus, at this point, we were unable to discriminate the individual contribution of these two activities to the overall efficacy of PARPi in *BRCA1*-mutated cells. The observation that the PARP1 L777P mutation enhanced the trapping activity of veliparib and olaparib without improving their PARylation inhibition potency, has provided for the first time, the opportunity to differentiate the two components of PARPi mechanism (Figs 7K, 8G, H and Supplementary Fig. S10G). The improved PARP1 trapping activities of veliparib and olaparib by the L777P mutation has been shown to translate into significantly improved efficacy of both the PARPi in *BRCA1*m cancer cells (Figs. 7L and 8A). Collectively, these observations provide important evidence that PARP1 trapping is a key mechanism in driving the effectiveness of PARPi in *BRCA1*-mutated cancer cells.

In light of recent and past research, it is relevant to consider the characteristics that define a potent and versatile PARPi. Primarily, targeting only PARP1, and not PARP2, is sufficient for efficacy in HRR-deficient cancer cells [23, 24, 27, 30]. Furthermore, we suggest that an inhibitor demonstrating significant PARP1 trapping capability along with potent PARylation inhibition will be the most effective as a single agent for cancers characterised by HRR deficiency. There is a caveat to this last statement though, which is that extreme PARP1 trapping activity may lead to undesirable effects in the form of normal tissue cytotoxicity in HRR-proficient cells [29, 30], potentially eroding the therapeutic index of PARPi. Therefore, to avoid cytotoxicity in HRR-proficient cells, PARP1 trapping should selectively occur in HRR-deficient cancer cells, as observed with saruparib (Fig. 2D). Mechanistically, our results suggest that the selective cytotoxicity of PARPi in HRR-deficient cells is achieved thanks to an optimal combination of (i) higher levels of endogenous DNA damage present in HRR-deficient cancer cells during S-phase, (ii) the PARP1 trapping potency of the PARPi, and (iii) the insufficient DNA repair capability of HRR-deficient cells (Fig. 8K).

One interesting feature of our work, with potential implications for therapeutic strategies, is the hyperactivity conferred by the PARP1 L777P mutation. We found that cells expressing this hyperactive PARP1 mutant are particularly sensitive to PARG or NAMPT inhibition, indicating pronounced dependency of these cells on NAD^+^ (Fig. 5 and Supplementary Fig. S7). It is worth noting that cells harbouring an allele coding a hyperactive PARP1 variant displayed elevated PARylation activity only after acute PARG inhibition, and such inhibition was toxic in the long-term (Fig. 5H–J). These observations suggest that PARG buffers high and toxic PAR levels produced by a hyperactive PARP1 variant, allowing cellular adaptation and survival. To date, the frequency of *PARP1* mutations that result in PARP1 hyperactivation within cancer populations remains unclear. Nonetheless, prior research has indicated that XRCC1 deficiency can lead to PARP1 hyperactivation, and notably, lack of XRCC1 expression has been reported in tumours, including triple-negative breast cancers [88, 89]. Consistent with this, XRCC1 deficiency has been observed to hypersensitise cells to PARG inhibition [90]. PARG and NAMPT inhibitors could therefore represent therapeutic opportunities for cancers displaying PARP1 hyperactivity, including XRCC1-deficient cancers.

The hyperactivity of the PARP1 L777P mutant could be explained thanks to our structural analysis of this mutation (Fig. 5). We found that this mutation enhances the dynamicity of the auto-inhibitory HD domain of PARP1. This includes the helix αF described by the seminal work of the Black and Pasqual teams, which detailed how PARP1 structural alterations upon DNA binding involve local unfolding of the helix αF [11]. Unfolding of this same helix is also observed during the PARPi-mediated trapping of PARP1 at a DNA lesion [14]. Considering these findings, we propose that the observed pro-trapping effect of the L777P mutation could be attributed to unfolding of the αF helix, which engages the mutant PARP1 into a conformation with high affinity for DNA lesions (Figs 6G, H, and 9E).

Through our HDX-MS analysis, we identified the binding signature of saruparib on PARP1, offering new insights into the conformational changes induced by saruparib binding (Fig. 6A). The same binding signature of saruparib was also observed in the PARP1 L777P protein, confirming the ability of saruparib to bind this mutant (Fig. 6D). We also determined that saruparib induces a reduction in the conformational dynamics of the helix αF of the HD domain of PARP1 L777P (Fig. 6D), that is associated with the release of this PARP1 mutant from DNA lesions upon saruparib binding (Fig. 6I). We propose that the ability of saruparib to promote the release of PARP1 L777P may be due to its pro-stabilisation effect on the helix αF (Fig. 9E). This hypothesis aligns with previous findings that suggest PARPi promoting the release of PARP1 tend to stabilise the αF helix of the HD domain [14].

Our results suggest that allosteric changes provide a structural explanation for the observed pro-trapping effect of the L777P mutation, as well as the respective release or trapping activities of saruparib or olaparib on the PARP1 L777P mutant (Fig. 9E). However, PARPi-induced destabilisation of the HD subdomain alone does not seem to account for the potent trapping activity of veliparib on PARP1 L777P, nor the trapping activities on the wild-type PARP1 by saruparib, olaparib, or veliparib (Fig. 9E). Although our structural analyses were limited to the CAT domain of PARP1, which prevents assessment of the contributions from other PARP1 domains, previous studies suggest that the dissociation rates of the different PARPi may also govern their trapping activities on PARP1 [91, 92]. Both allostery and dissociation rate could act in concert to govern the PARP1 trapping activity of a PARPi.

However, the dissociation rate does not explain the changes in trapping activities of veliparib and saruparib on PARP1 L777P, since there is no change in the dissociation rates or affinities of these compounds to the PARP1 mutant compared to wild-type (Supplementary Fig. S8). Furthermore, the stabilisation effect by veliparib on the HD domain of PARP1 L777P (Fig. 9C) is unexpected for a potent PARP1 trapper. Work from Black and Pascal teams suggests that the HD domain stabilisation facilitates PARP1 release from DNA lesions, and recently, Kanev *et al.* reported that saruparib promotes the chromatin retention of PARP1 by accelerating the binding-release cycles of PARP1 at DNA lesions [14, 93]. Our data show that the trapper saruparib has a moderate stabilisation effect on the HD domain of wild-type PARP1 (Fig. 6A). This structural change could induce the acceleration of the PARP1 binding-release cycles and retention at DNA lesions suggested by Kanev and colleagues. Thus, veliparib may induce trapping of PARP1 L777P via a similar mechanism (Fig. 9E). Altogether, we propose that PARP1 trapping is a dynamic multi-molecular mechanism that can occur via both structural changes: (i) destabilisation of the HD domain, which promotes stable binding of PARP1 to DNA lesions (candidate examples: apo L777P PARP1, olaparib-bound L777P PARP1); (ii) moderate stabilisation of the HD domain, which promotes rapid binding-release cycles of PARP1 at DNA lesions (candidate examples: saruparib-bound wild-type PARP1, veliparib-bound L777P PARP1) (Fig. 9E).

The notion that PARP1 trapping is a key mechanism driving the effectiveness of PARPi in BRCAm cancer cells is well-supported by the scientific literature [8, 9, 26, 27, 30] but has more recently been questioned [29, 87]. The work described here includes an original genetic demonstration of the importance of PARP trapping in the single agent activity of PARPi in HRR-deficient cancer cells, while introducing important insights that can inform the development of future PARPi-associated therapeutic strategies.

## Supplementary Material

gkaf1398_Supplemental_Files

## Data Availability

BioSample accessions: SAMN47378027, SAMN47378028, SAMN47378029 Accession PRJNA1235993 (https://dataview.ncbi.nlm.nih.gov/object/PRJNA1235993).
