# Supplementary material for: Genetic evidence for PARP1 trapping as a driver of PARP inhibitor efficacy in BRCA mutant cancer cells
Source: Nucleic Acids Res. 2025 Dec 29;53(22):gkaf1398. doi: 10.1093/nar/gkaf1398 (PMC12746104; doi:10.1093/nar/gkaf1398)
Supplement: gkaf1398_Supplemental_Files [file gkaf1398_supplemental_files.zip › Supplementary Figures Ribeiro et al.pdf]

Supplementary Information for:

Genetic Evidence for PARP1 Trapping as a Driver of PARP Inhibitor Efficacy in *BRCAm* cancer cells

Jonathan Ribeiro, Kjetil Hansen, Lotte van Beek, Christopher Stubbs, Joan Frigola, Sara Talbot, Sabrina Bentouati, James Hall, Paul W.G. Wijnhoven, Josh Armenia, Marianne Schimpl, Josep V Forment, Mark Albertella, Mark J. O'Connor, Giuditta Illuzzi

This PDF file includes:
Supplementary Figures S1 to 14.

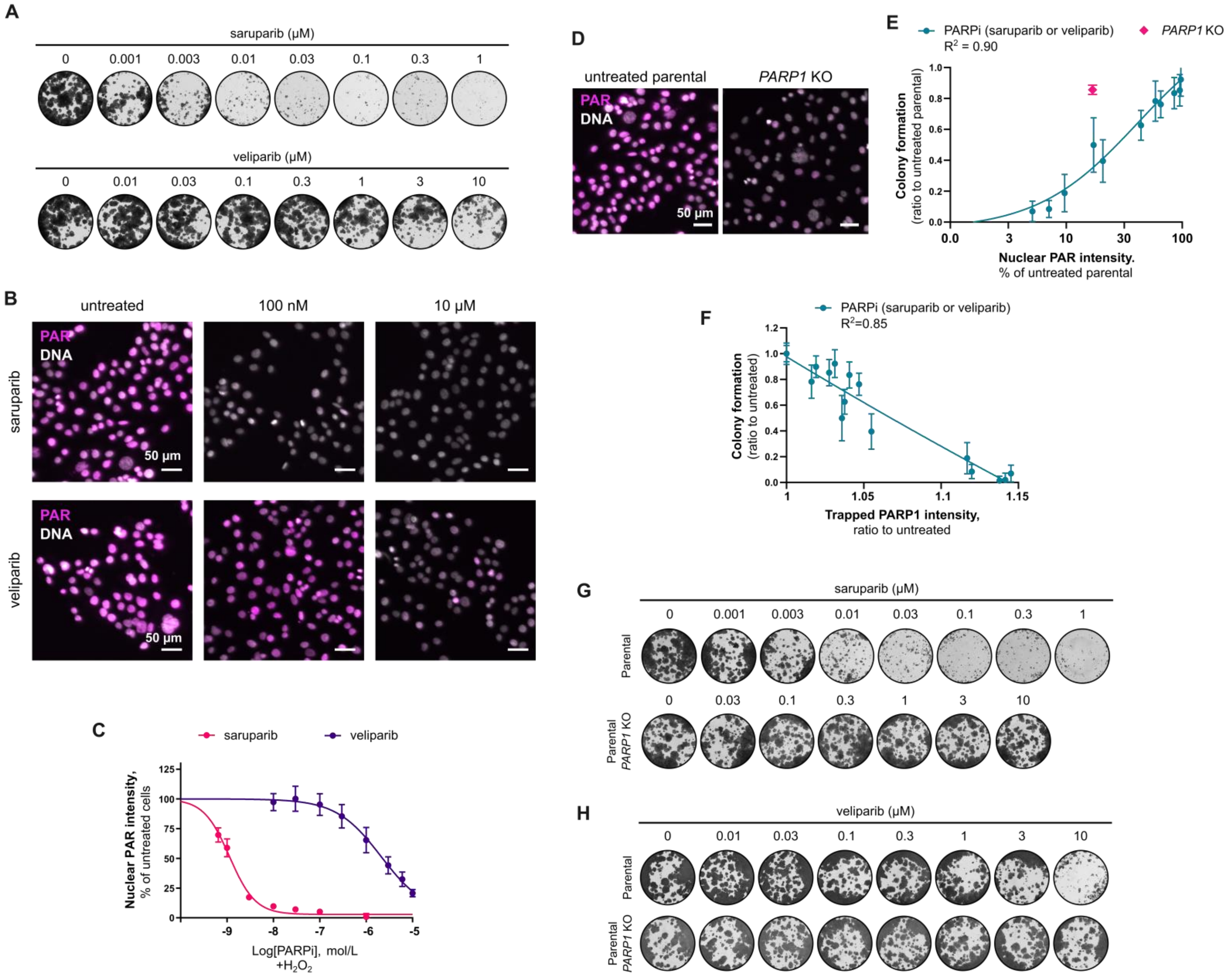

Supplementary Figure S1

Supplementary Figure S1: Importance of PARP1 for the efficacy of PARPi, related to Figure 1.

(A) Representative images of colony formation assays performed with SUM149PT cells treated with saruparib or veliparib for 9 days. (B, C) PARylation inhibition assay in SUM149PT cells treated with saruparib or veliparib and the DNA damaging agent H_2O_2 (10 mM). (D) Representative images of PARylation immunofluorescence performed with parental SUM149PT cells or *PARP1* KO SUM149PT cells, treated with the DNA damaging agent H_2O_2 (10 mM). (E) Correlation between colony formation capacity and nuclear PAR intensity in parental SUM149PT cells treated with PARPi (saruparib or veliparib) or in untreated *PARP1* KO SUM149PT cells. (F) Correlation between colony formation capacity and PARP1 trapping in parental SUM149PT cells treated with PARPi (saruparib or veliparib) and 0.005% MMS. (G,H) Representative images of colony formation assays performed with parental and *PARP1* KO SUM149PT cells treated with saruparib (G) or veliparib (H) for 9 days. In every plots, data shown are the means of at least three independent experiments, and error bars indicate ±SD.

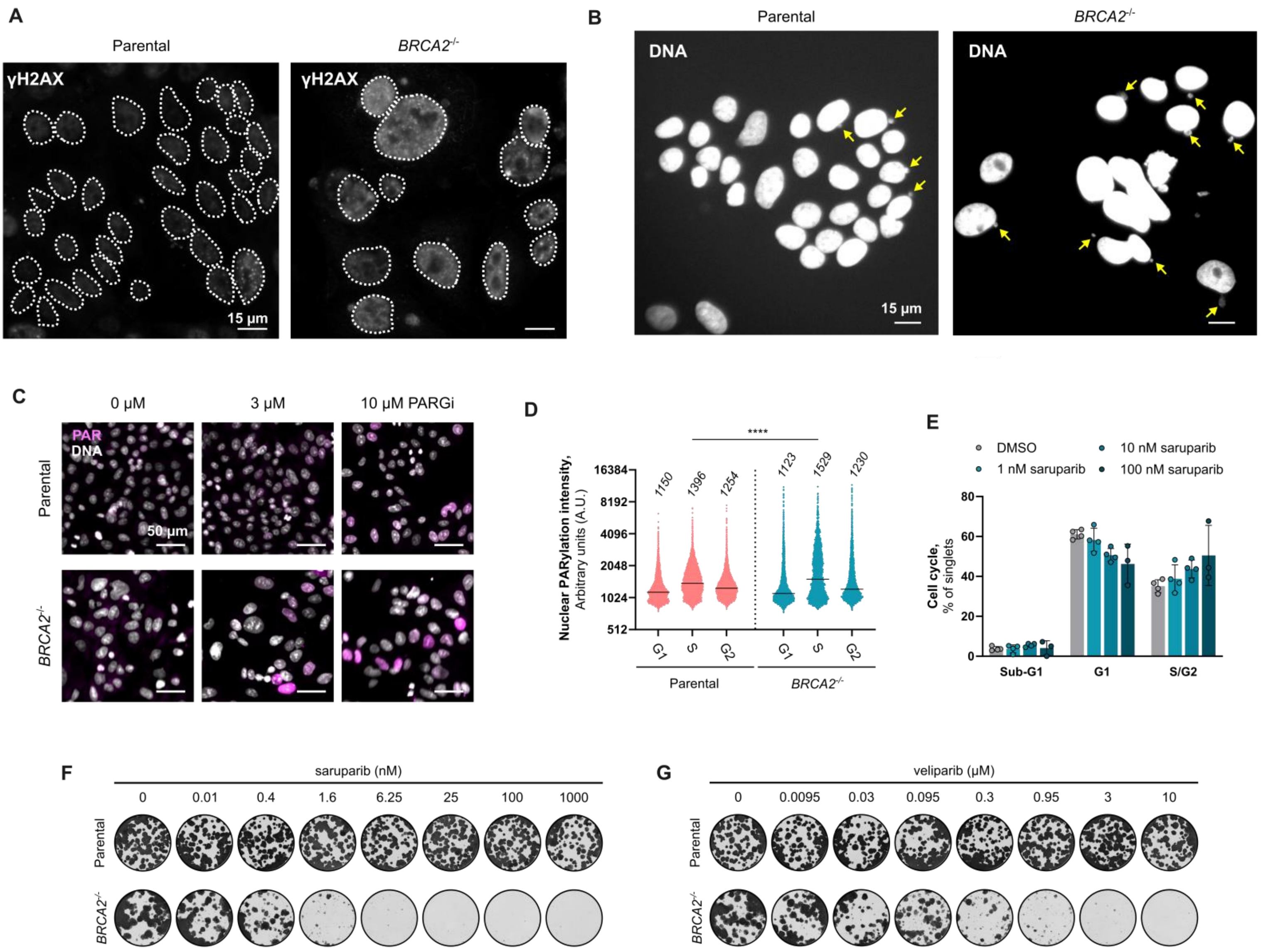

Supplementary Figure S2

Supplementary Figure S2: Profiling of DNA damage, micronuclei and PARP activity in *BRCA2*-deficient cells, related to Figure 2.

(A) Representative immunofluorescence images of γH2AX in untreated parental and *BRCA2*$^{-/-}$ DLD-1 cells. Nuclei are delineated with dashed circles. (B) Representative images of DAPI-stained micronuclei in untreated parental and *BRCA2*$^{-/-}$ DLD-1 cells. Yellow arrows indicate micronuclei. (C) Representative immunofluorescence images of PAR in parental and *BRCA2*$^{-/-}$ DLD-1 cells, after 1h exposure to a PARG inhibitor (PARGi, PDD 00017273). (D) Quantifications of immunofluorescence analysis of PAR in parental and *BRCA2*$^{-/-}$ DLD-1 cells, after 1h exposure to a PARG inhibitor (10 µM PARGi, PDD 00017273). Cells are segregated into cell cycle phases using their DNA content (DAPI intensities). A least 3800 cells are displayed per cell cycle phase. Indicated values are the medians of the PAR intensities per cell cycle phase. P values calculated using a Mann-Whitney test. (E) Cell cycle distribution in in parental and *BRCA2*$^{-/-}$ DLD-1 cells treated with saruparib for 72h. Data shown are the means of two independent experiments, and error bars indicate ±SD. (F,G) Representative images of colony formation assays performed with parental and *BRCA2*-deficient DLD1 cells treated with saruparib (F) or veliparib (G).

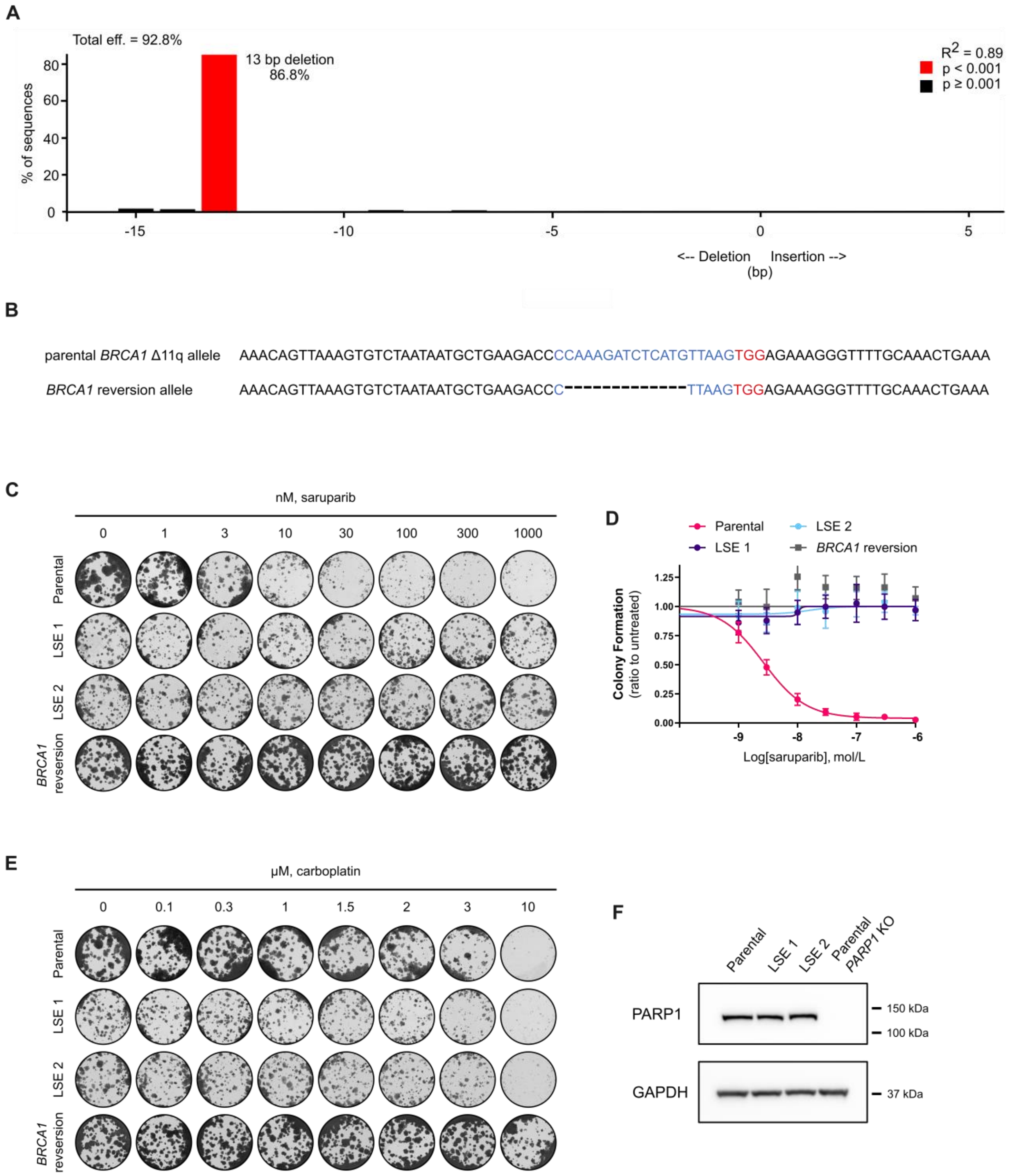

Supplementary Figure S3

Supplementary Figure S3: saruparib and carboplatin effects on the cell survival of LSE cells and expression of PARP1 in these cells, related to Figure 3.

(A) Indel spectrum obtained after TIDE analysis of *BRCA1* reversion SUM149PT cells. (B) Frameshift mutation in *BRCA1* reversion SUM149PT cells. The gRNA targeting *BRCA1* is displayed in blue and PAM in red. (C) Representative images and (D) quantifications of colony formation assays performed with parental, LSE and *BRCA1* reversion SUM149PT cells treated with saruparib for 9 days. Data shown are the means of at least three independent experiments, and error bars indicate ±SD. (E) Representative images of colony formation assays performed with parental, LSE and *BRCA1* reversion SUM149PT cells treated with carboplatin for 9 days. (F) PARP1 protein expression assessed by western blot in parental, *PARP1* KO and LSE SUM149PT cells; GAPDH is used as loading control. Forty micrograms of proteins were loaded per well.

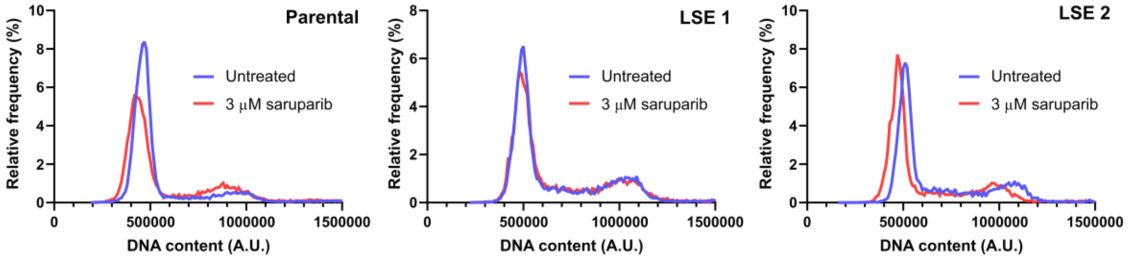

Supplementary Figure S4

Supplementary Figure S4: Cell cycle profiles of LSE cells after 3 days exposure to saruparib, related to Figure 4.

Cell cycle distribution in SUM149PT parental cells or the LSE clones 1 and 2 treated with saruparib for 3 days.

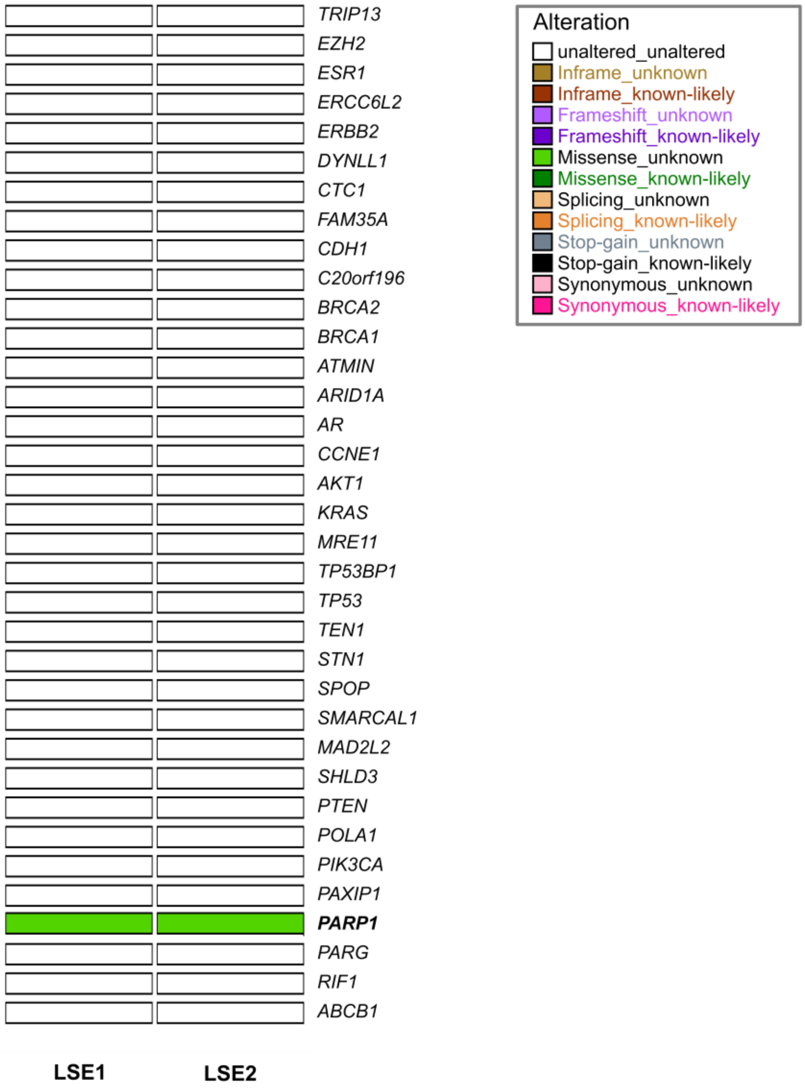

Supplementary Figure S5

Supplementary Figure S5: Whole exome sequencing identifies PARP1 L777P in LSE cells, related to Figure 5.

Assessment of mutations in LSE clones in a manually curated set of genes associated with known resistance mechanisms to PARPi. The missense mutation identified in LSE clones is PARP1 L777P (codon change: c.2330T>C).

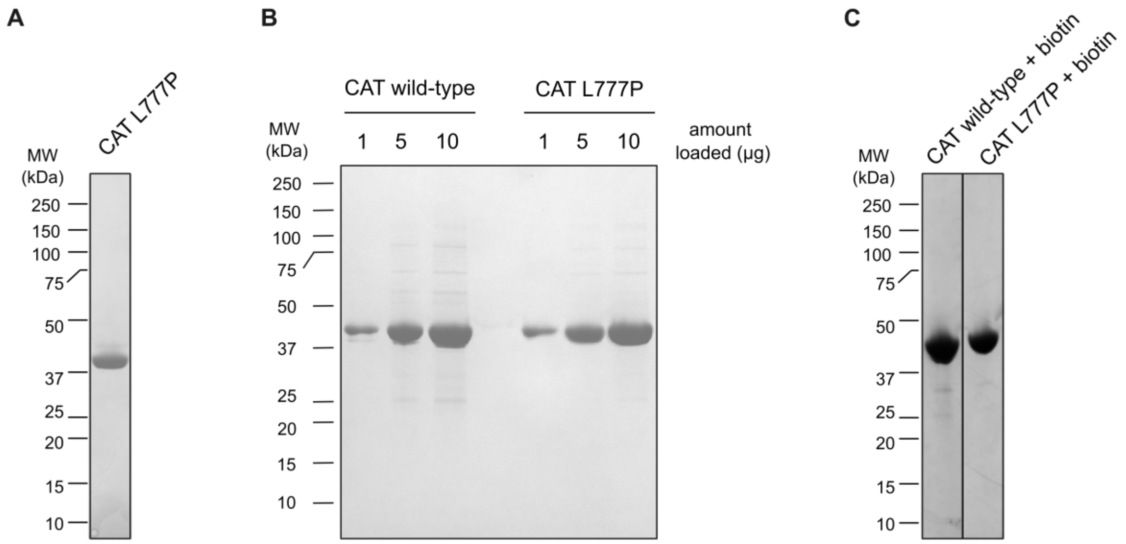

Supplementary Figure S6

Supplementary Figure S6: Purification of CAT PARP1 wild-type and L777P, related to Figure 5.

(A) Coomassie staining of purified CAT PARP1 L777P (residues 662-1011) analysed by SDS-PAGE. (B) Coomassie staining of purified CAT PARP1 wild-type or L777P (residues 662-1011) analysed by SDS-PAGE. (C) Coomassie staining of purified CAT PARP1 wild-type or L777P (residues 662-1011) analysed by SDS-PAGE after biotinylation.

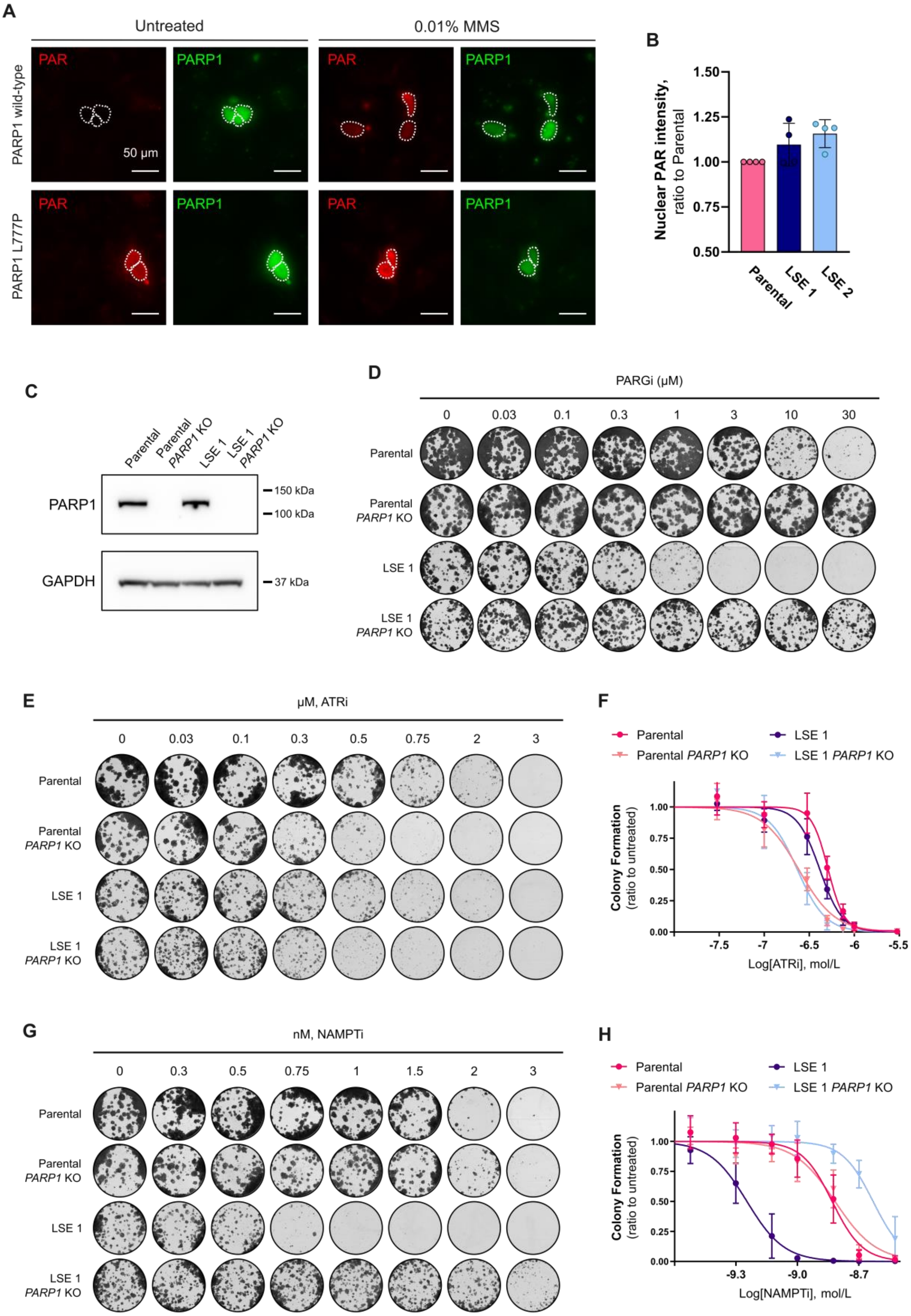

Supplementary Figure S7

Supplementary Figure S7: Characterisation of the hyperactivity of PARP1 L777P, related to Figure 5.

(A) Representative immunofluorescence images of A549 *PARP1/PARP2* KO cells transiently overexpressing wild-type or L777P mutant PARP1, untreated or treated with 0.01% MMS. PARP1 expressing nuclei (green) are delineated with dashed circles and PAR signal is displayed in red. (B) Quantification by immunofluorescence of the nuclear PAR signal in untreated parental and LSE SUM149PT cells. (C) PARP1 protein expression assessed by western blot in parental SUM149PT cells, LSE clone 1 and their respective *PARP1* KOs. GAPDH is used as loading control. Forty micrograms of proteins were loaded per well. (D) Representative images of colony formation assays performed with parental SUM149PT cells, the clone LSE1 and their respective *PARP1* KOs after 9 days exposure to PARGi (PDD 00017273). (E) Representative images and (F) quantification of colony formation assays performed with parental SUM149PT cells, the clone LSE1 and their respective *PARP1* KOs after 9 days exposure to the ATR inhibitor ceralasertib. (G) Representative images and (H) quantification of colony formation assays performed with parental SUM149PT cells, the clone LSE1 and their respective *PARP1* KOs after 9 days exposure to the NAMPT inhibitor CHS-828. In all plots, data shown are the means of at least three independent experiments, and error bars indicate ±SD.

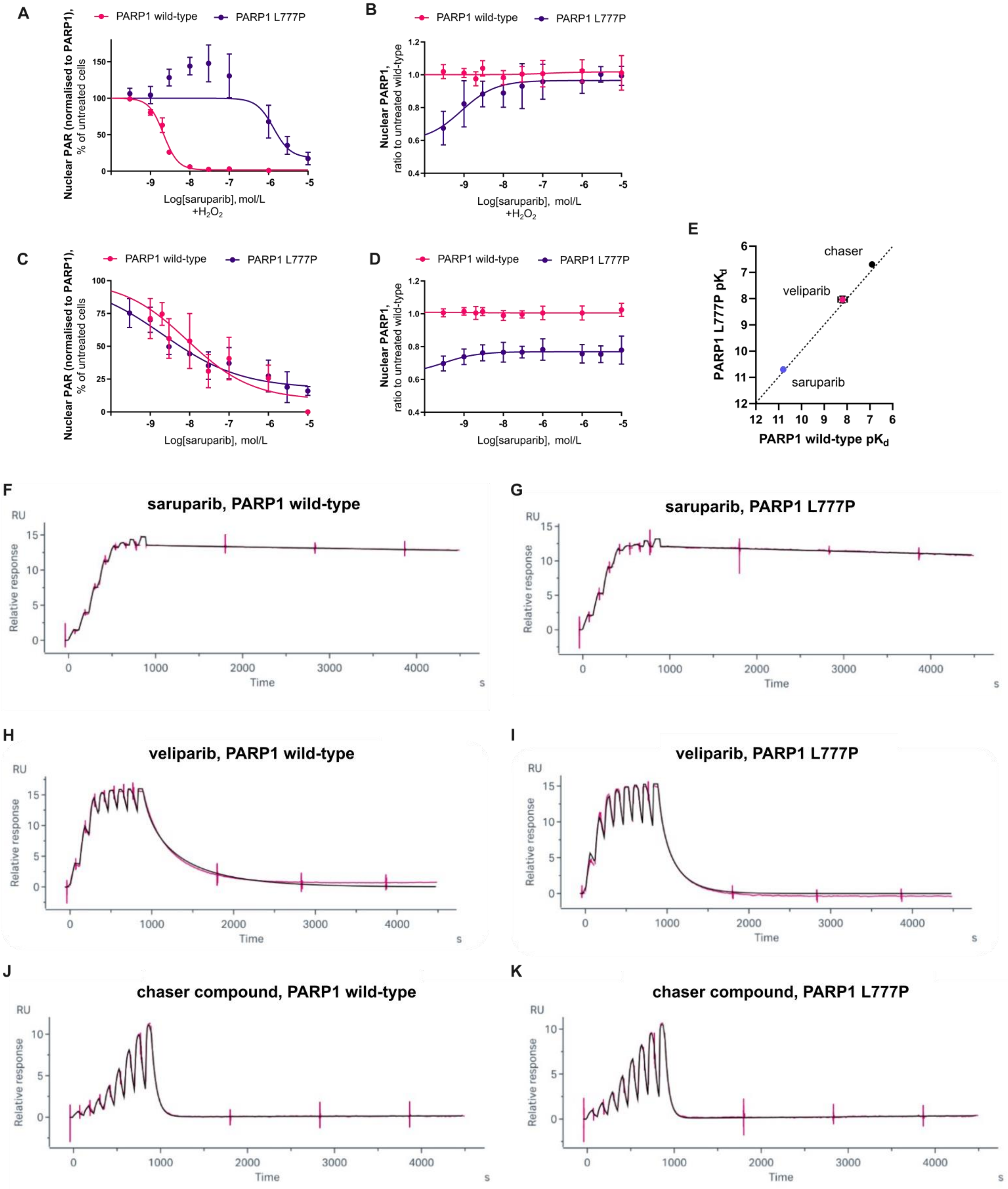

Supplementary Figure S8

Supplementary Figure S8: Biochemical characterisation of saruparib effects on PARP1 L777P and affinities of PARPi to PARP1 L777P, related to Figure 6.

(A) PARylation inhibition assay with SUM149PT *PARP1* KO cells transiently overexpressing wild-type PARP1 or PARP1 L777P and treated with saruparib and 10 mM H_2O_2. (B) PARP1 levels in PARylation assay displayed in (A). (C) PARylation inhibition assay with SUM149PT *PARP1* KO cells transiently overexpressing wild-type PARP1 or PARP1 L777P, treated with saruparib, and not exposed to other DNA damaging agent. (D) PARP1 levels in PARylation assay displayed in (C). Data shown in (A) (B) (C) and (D) are the means of two independent experiments, and error bars indicate ±SD. (E) Affinities of saruparib, veliparib and the chaser compound to PARP1 WT and L777P, measured by Surface Plasmon Resonance (SPR). (F,G) SPR sensorgrams for binding of saruparib to (F) PARP1 WT and (G) PARP1 L777P. (H,I) SPR sensorgrams for binding of veliparib to (H) PARP1 WT and (I) PARP1 L777P. (J,K) SPR sensorgrams for binding of the chaser compound to (J) PARP1 WT and (K) PARP1 L777P. Recorded data in magenta, fitted model in black.

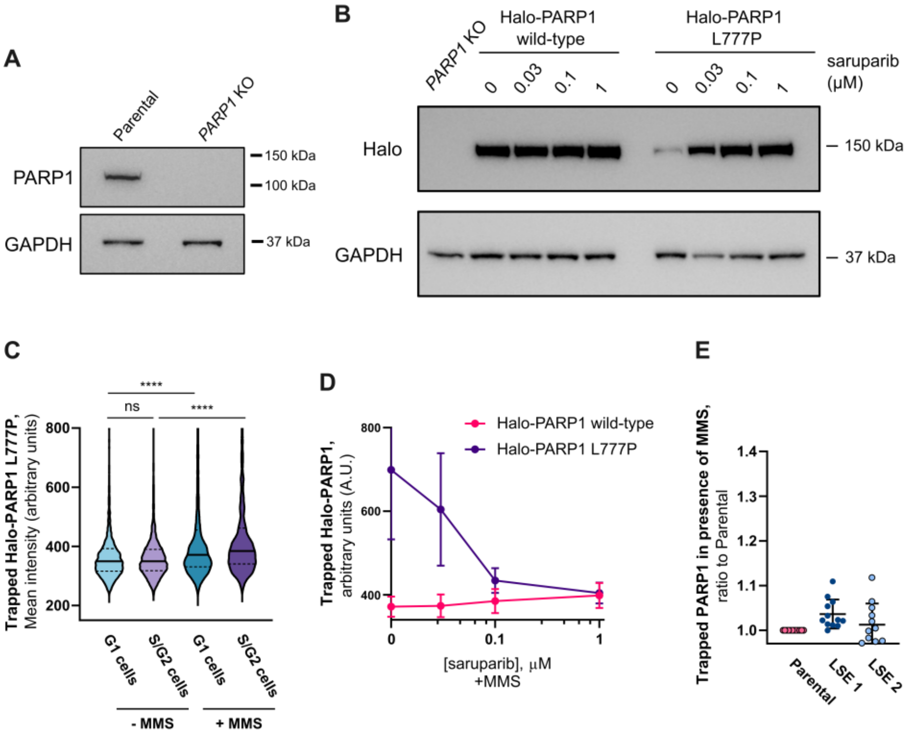

Supplementary Figure S9

Supplementary Figure S9: Characterisation of saruparib effects on the trapping of PARP1 L777P, related to Figure 6.

(A) PARP1 protein expression assessed by western blot in parental and *PARP1* KO HEK293T cells. GAPDH is used as loading control. Forty micrograms of proteins were loaded per well. (B) Halo-PARP1 expression assessed by western-blot in HEK293T *PARP1* KO cells transiently overexpressing wild-type or mutant Halo-PARP1 and exposed for 16h with saruparib. GAPDH is used as loading control. (C) Quantification of trapped Halo-PARP1 L777P in G1 or S/G2 *PARP1* KO HEK293T cells. A least 1800 cells are displayed per cell cycle phase. Data shown are the medians, and error bars indicate quartiles. P values calculated using a Mann-Whitney test. (D) Effect of saruparib on the trapping of wild-type or L777P Halo-PARP1 (absolute fluorescence intensities, arbitrary units). Data shown are the means of three independent experiments, error bars indicate ±SD. (E) Effect of 0.005% MMS treatment on the trapping of PARP1 in LSE SUM149PT cells (ratio to MMS-treated Parental SUM149PT cells). Data shown are twelve experimental repeats from three independent experiments, and the means of three independent experiments, and error bars indicate ±SD.

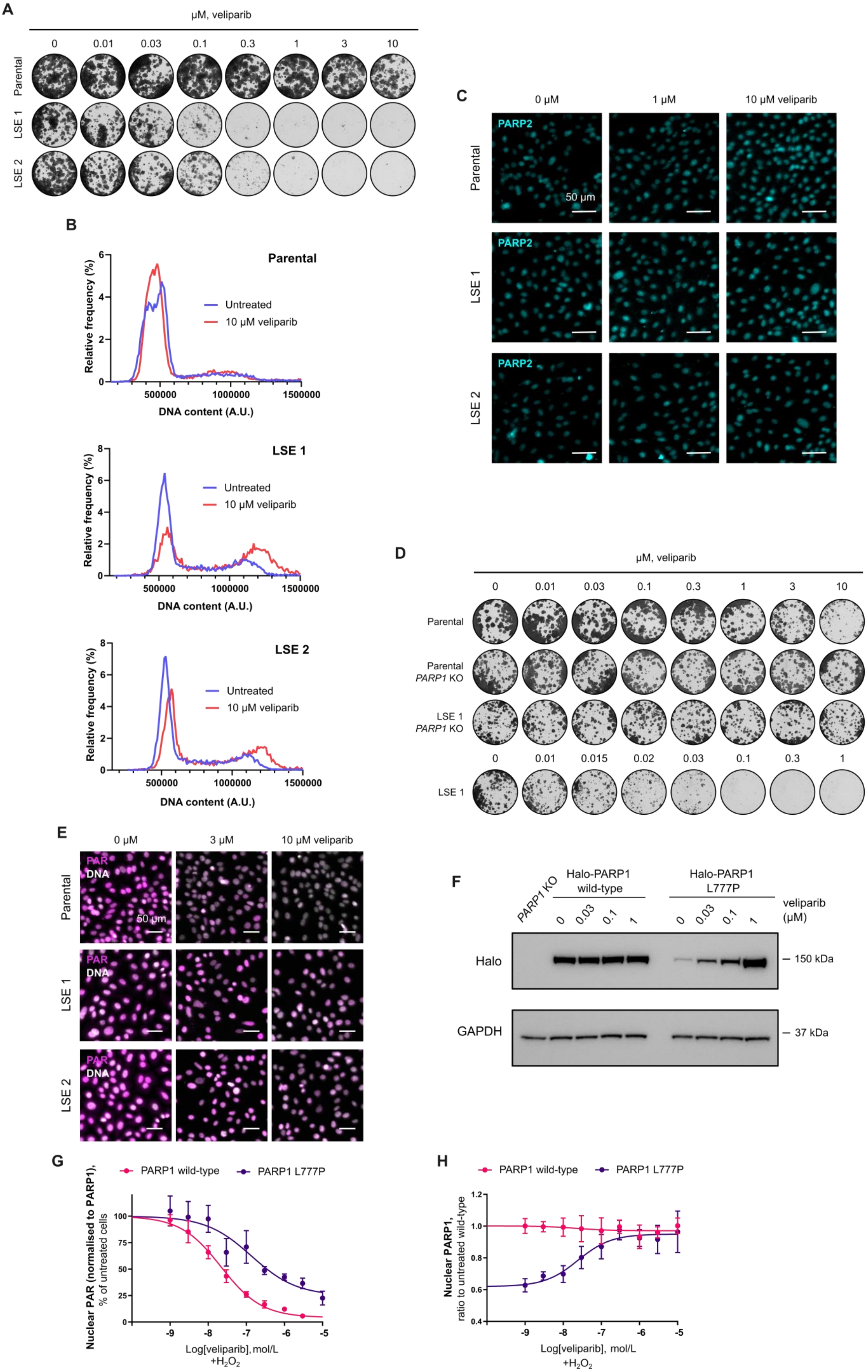

Supplementary Figure S10

Supplementary Figure S10: Characterisation of the sensitivity of LSE cells to veliparib, related to Figure 7.

(A) Representative images of colony formation assays performed with parental and LSE SUM149PT cells treated with veliparib for 9 days. (B) Cell cycle distribution in parental and LSE SUM149PT cells treated with veliparib for 3 days. (C) Representative immunofluorescence images of PARP2 trapping in parental and LSE SUM149PT cells, after treatment with veliparib and 0.005% MMS. (D) Representative images of colony formation assays performed with parental SUM149PT cells, the clone LSE1 and their respective *PARP1* KOs after 9 days exposure to veliparib. (E) Immunofluorescence-based PARylation inhibition assay in parental and LSE SUM149PT cells treated with veliparib and 10 mM H_2O_2. (F) Halo-PARP1 expression assessed by western-blot in HEK293T *PARP1* KO cells transiently overexpressing wild-type or mutant Halo-PARP1 and exposed for 16h with veliparib. GAPDH is used as loading control. Forty micrograms of proteins were loaded per well. (G) PARylation inhibition assay with SUM149PT *PARP1* KO cells transiently overexpressing wild-type PARP1 or PARP1 L777P and treated with veliparib and 10 mM H_2O_2. Data shown are the means of two independent experiments, and error bars indicate ±SD. (H) PARP1 levels in PARylation assay displayed in (G). Data shown are the means of two independent experiments, and error bars indicate ±SD.

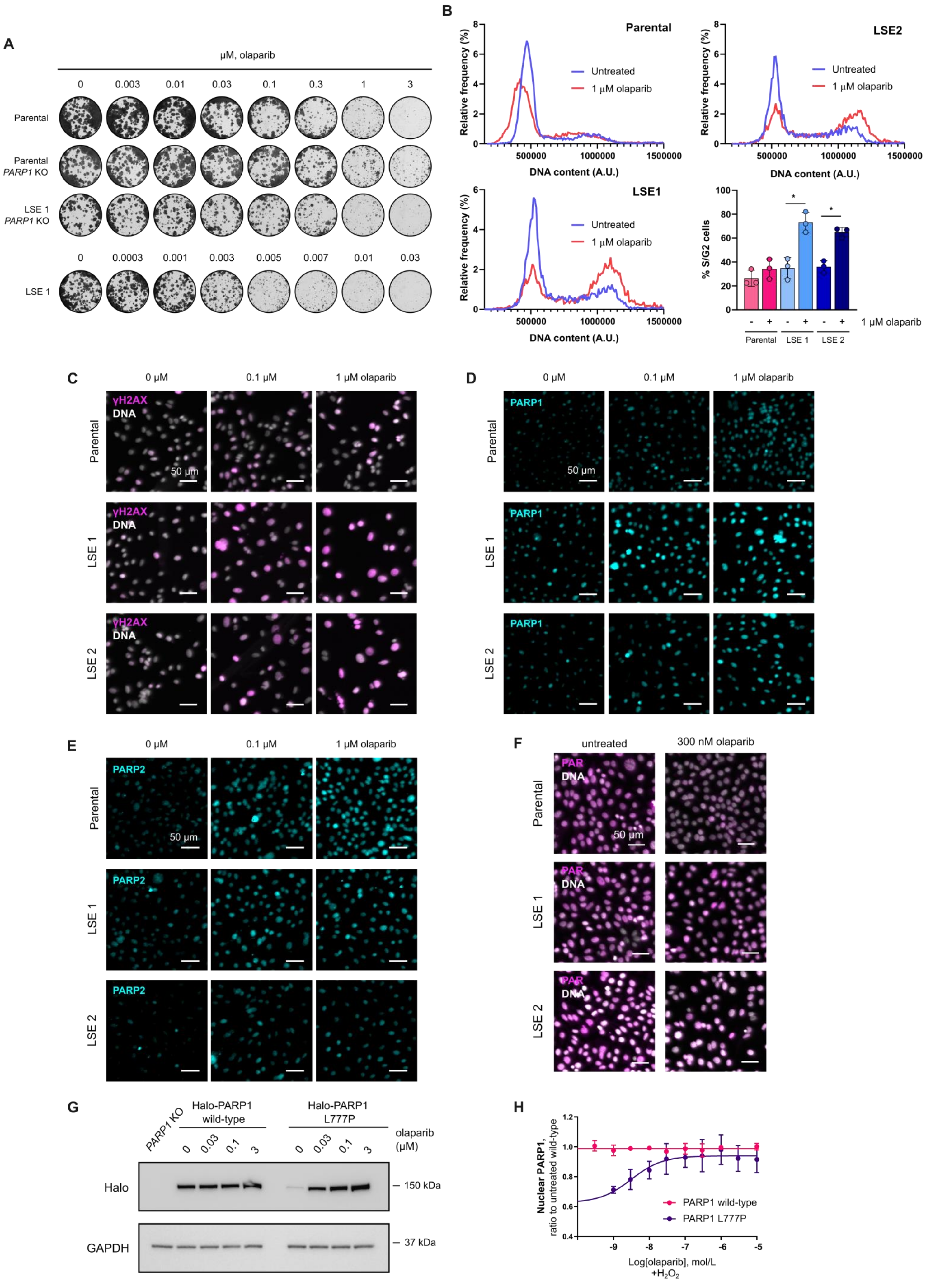

Supplementary Figure S11

Supplementary Figure S11: Characterisation of the sensitivity of LSE cells to olaparib, related to Figure 8.

(A) Representative images of colony formation assays performed with parental SUM149PT cells, the clone LSE1 and their respective *PARP1* KOs after 9 days exposure to olaparib. (B) Cell cycle analysis in parental and LSE SUM149PT cells, after 72 h treatment with olaparib. Data shown are the means of three independent experiments, and error bars indicate ±SD. (C) Representative immunofluorescence images of the DNA damage marker γH2AX in parental and LSE SUM149PT cells, after 72 h treatment with olaparib. (D) Representative immunofluorescence images of PARP1 trapping in parental and LSE SUM149PT cells, after treatment with olaparib and 0.005% MMS. (E) Representative immunofluorescence images of PARP2 trapping in parental and LSE SUM149PT cells, after treatment with olaparib and 0.005% MMS. (F) Representative images of the immunofluorescence-based PARylation inhibition assay in parental and LSE SUM149PT cells treated with olaparib and 10 mM H_2O_2. (G) Halo-PARP1 expression assessed by western-blot in HEK293T *PARP1* KO cells transiently overexpressing wild-type or mutant Halo-PARP1 and exposed for 16h with olaparib. GAPDH is used as loading control. Thirty micrograms of proteins were loaded per well. (H) PARP1 levels in PARylation assay displayed in Figure 8H. Data shown are the means of two independent experiments, and error bars indicate ±SD.

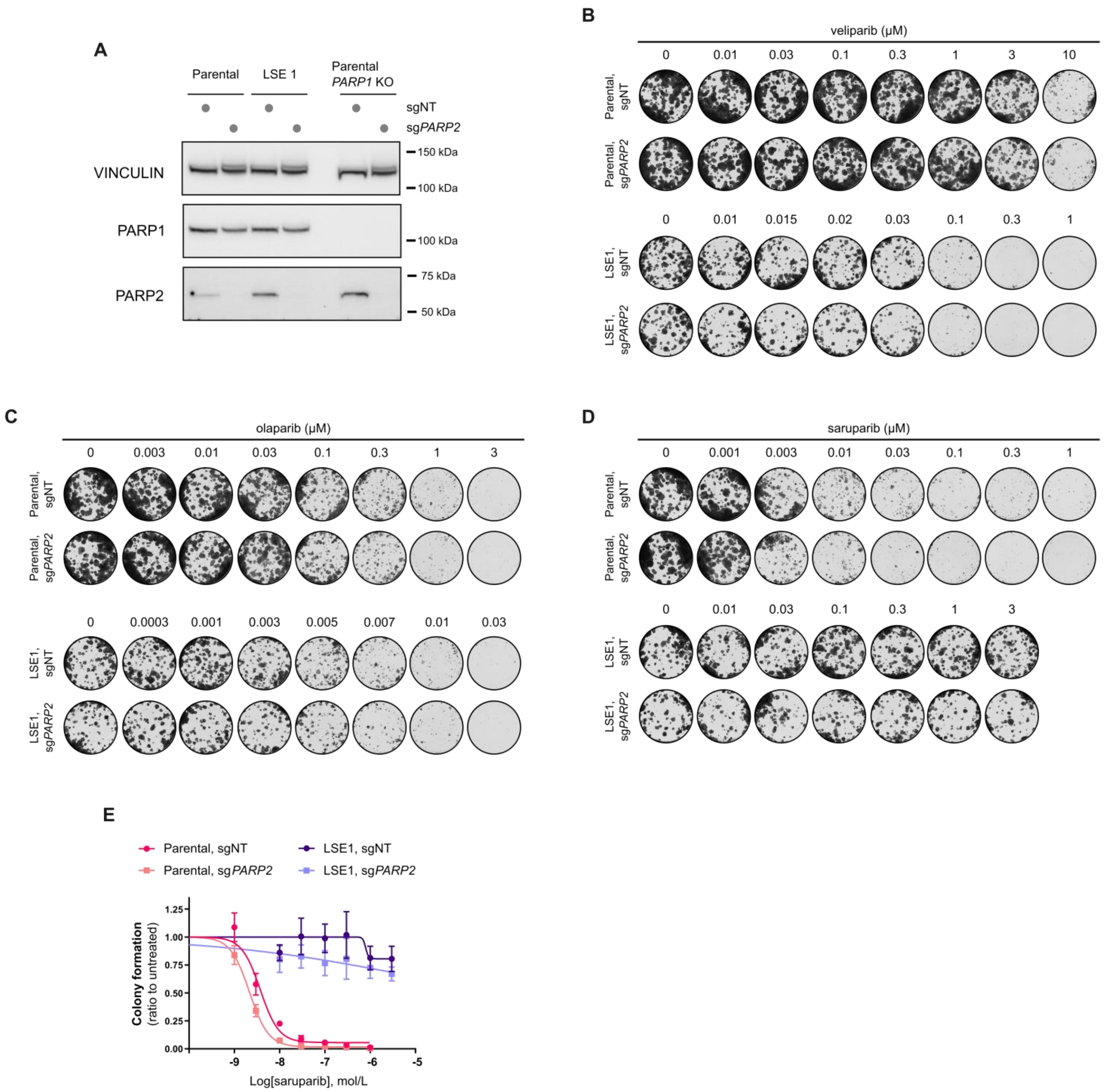

Supplementary Figure S12

Supplementary Figure S12: Role of PARP2 in the efficacies of veliparib, olaparib and saruparib, related to Figure 8.

(A) PARP2 protein expression assessed by western blot in parental SUM149PT cells or the clone LSE1, eight days after transfection with Cas9 and a non-targeting sgRNA (sgNT) or with sgRNA targeting *PARP2* (sg*PARP2*). VINCULIN is used as loading control. Sixty micrograms of proteins were loaded per well. (B,C) Representative images of colony formation assays performed with parental SUM149PT cells or the clone LSE1 transfected with a non-targeting sgRNA (sgNT) or with sgRNA targeting *PARP2* (sg*PARP2*), and after 9 days exposure to veliparib (B) or olaparib (C). (D) Representative images and (E) quantification of colony formation assays performed with parental SUM149PT cells or the clone LSE1 transfected with a non-targeting sgRNA (sgNT) or with sgRNA targeting *PARP2* (sg*PARP2*), and after 9 days exposure to saruparib. Data shown are the means of two independent experiments, and error bars indicate ±SD.

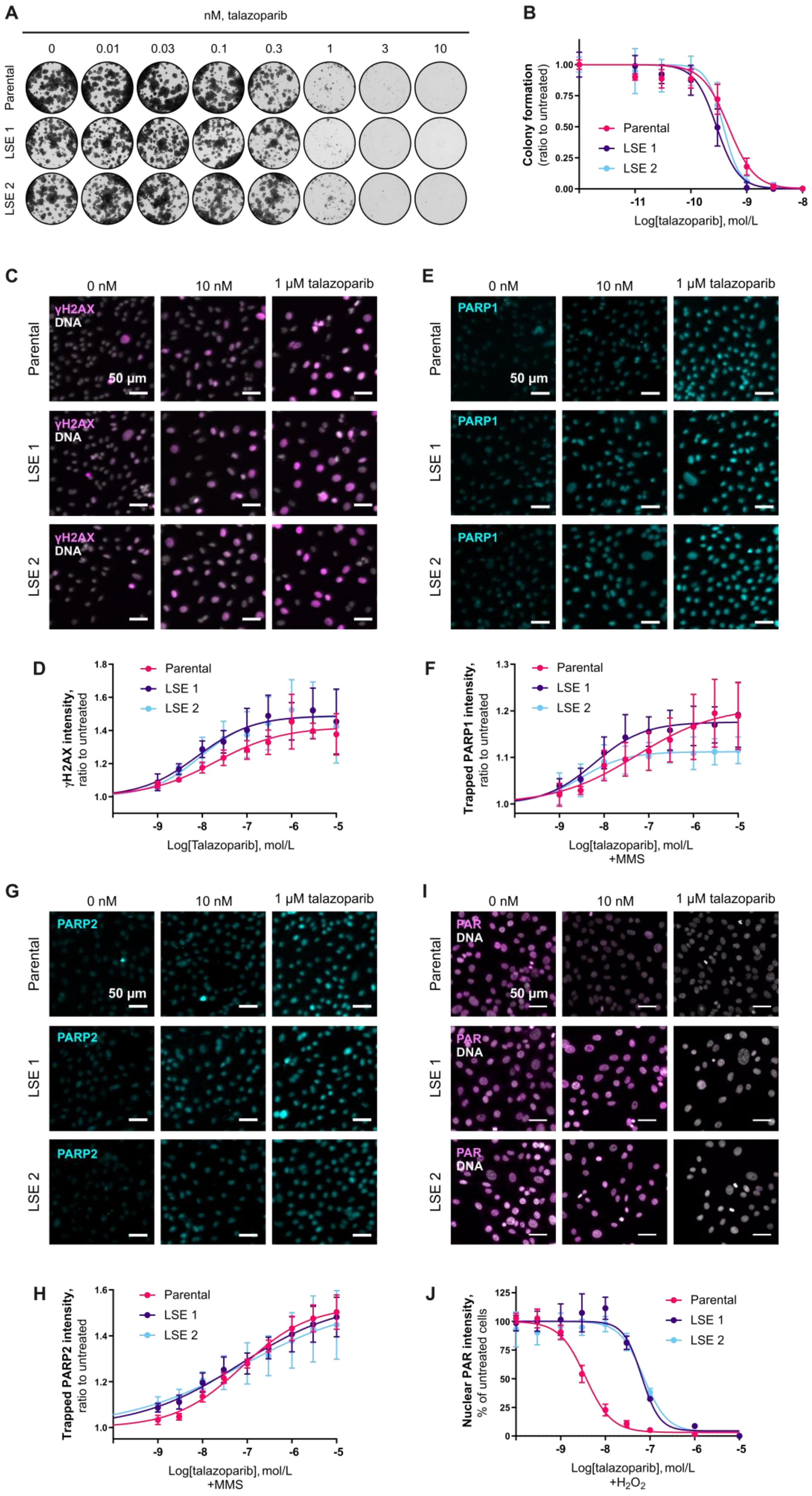

Supplementary Figure S13

Supplementary Figure S13: Characterisation of the mechanism of action of talazoparib in LSE cells.

(A) Representative images and (B) quantifications of colony formation assays performed with parental SUM149PT cells and the LSE clones 1 and 2 treated with talazoparib for 9 days. (C) Representative immunofluorescence images and (D) quantifications of immunofluorescence analysis of the DNA damage marker γH2AX in parental and LSE SUM149PT cells, after 72 h treatment with talazoparib. (E) Representative immunofluorescence images and (F) quantifications of immunofluorescence analysis of PARP1 trapping in parental and LSE SUM149PT cells, after treatment with talazoparib and 0.005% MMS. (G) Representative immunofluorescence images and (H) quantifications of immunofluorescence analysis of PARP2 trapping in parental and LSE SUM149PT cells, after treatment with talazoparib and 0.005% MMS. (I,J) Immunofluorescence-based PARylation inhibition assay in parental and LSE SUM149PT cells treated with talazoparib and 10 mM H_2O_2. Data shown are the means of three independent experiments, error bars indicate ±SD.

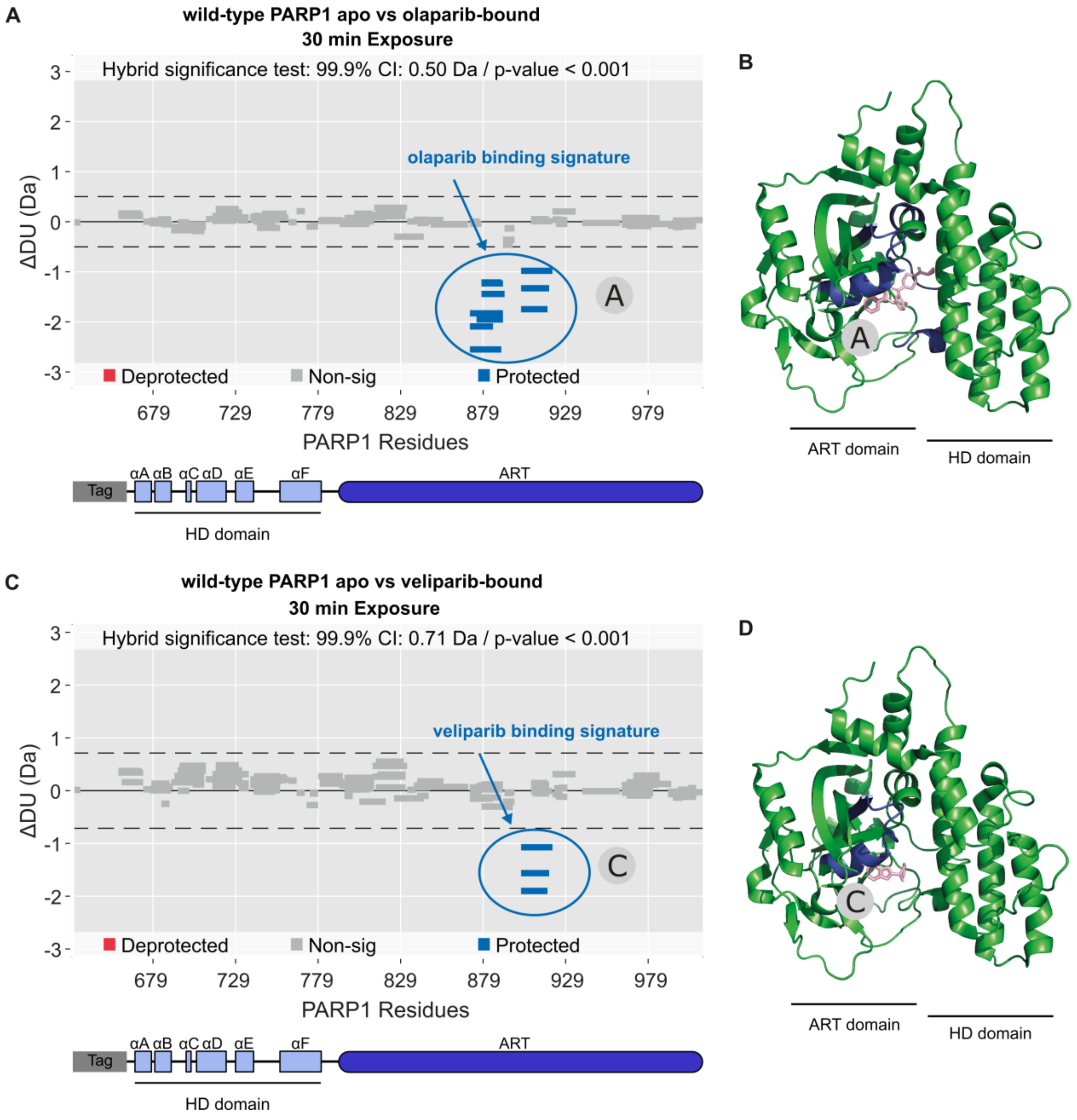

Supplementary Figure S14

Supplementary Figure S14: Binding signatures of olaparib and veliparib.

(A) Woods plot for the 30-minute timepoint showing the difference in deuterium uptake of peptides in between apo and olaparib-bound wild-type PARP1. A coverage of 92.69% was achieved across 150 peptides for the comparison between the unbound (apo) and olaparib-bound states of wild-type PARP1. The region of interest is called A and annotated in panel (B). (B) Crystal structure of PARP1 CAT domain with regions of significant olaparib-induced protection highlighted in blue (7AAD). Areas with no significance are displayed in green. (C) Woods plot for the 30-minute timepoint showing the difference in deuterium uptake of peptides in between apo and veliparib-bound wild-type PARP1. A coverage of 92.69% was achieved across 150 peptides for the comparison between the unbound (apo) and veliparib-bound states of wild-type PARP1. The region of interest is called C and annotated in panel (D). (D) Crystal structure of PARP1 CAT domain with regions of significant veliparib-induced protection highlighted in blue (7AAC). Areas with no significance are displayed in green. For wood plots, non-significant peptides are shown as grey bars. Significantly protected or deprotected peptides are shown as blue or red bars respectively. HDX-MS data are representative of two independent experiments.
